# Interplay of chromatin remodeling BAF complexes in mouse embryonic and epiblast stem cell conversion and maintenance

**DOI:** 10.1016/j.jbc.2024.108140

**Published:** 2024-12-25

**Authors:** Zhaoru Ma, Shuping Tan, Renhong Lu, Peixin Chen, Yukun Hu, Tenghui Yang, Hao Wu, Zhexin Zhu, Jiayi Guo, Xi Chen, Jian Yang, Wensheng Zhang, Ying Ye

**Affiliations:** 1 Cam-Su Genomic Resource Center, Medical College of Soochow University, Suzhou, China; 2 State Key Laboratory of Cardiology, Shanghai East Hospital, Tongji University School of Medicine, Shanghai, China; 3 Department of Biology, Southern University of Science and Technology, Shenzhen, China; 4 Hefei Comprehensive National Science Center, Institute of Health and Medicine, Heifei, China; 5 Research Center of Medical Science and Technology, Ningxia Medical University, Yinchuan, China; 6 Department of Clinical Pathobiology and Immunological Testing, School of Medical Laboratory, Qilu Medical University, Zibo, China

**Keywords:** ESCs, EpiSCs, BAF complex, LIF/STAT3, TGF-beta

## Abstract

Mouse embryonic stem cells (ESCs) and epiblast stem cells (EpiSCs) are pluripotent stem cells derived from preimplantation and postimplantation embryos, respectively. These cells are capable of interconversion through manipulation of key transcription factors and signaling pathways. While BRG1/BRM-associated factor (BAF) chromatin remodeling complexes are known to play crucial roles in ESC self-renewal and pluripotency, their roles in EpiSCs and their interconversion with ESCs remain unclear. This study demonstrates that the LIF/STAT3 and Wnt signaling pathways, in conjunction with canonical BAF (cBAF) and polycomb repressive complex two complexes, inhibit EpiSC gene expression, thereby preventing ESCs from converting to EpiSCs. Upon removal of LIF, the reduced LIF/STAT3 signaling lifts this inhibition, increasing TGF/nodal pathway activity. Subsequently, the cBAF complex facilitates ESC to EpiSC conversion by promoting EpiSC gene expression. Furthermore, unlike cBAF, inhibition of the ncBAF complex downregulates TGF-β signaling, thereby hindering both ESC to EpiSC conversion and EpiSC maintenance. Moreover, this study revealed the dual mechanisms, methylating histone or non-histone protein STAT3, by which polycomb repressive complex two components participate in the regulation of ESCs to EpiSCs. This research elucidates the interplay between distinct BAF complexes and specific signaling pathways in regulating the conversion and maintenance of ESCs and EpiSCs.

Embryonic stem cells (ESCs) and epiblast stem cells (EpiSCs) are pluripotent stem cells derived from the preimplantation blastocyst and post-implantation epiblast in early mouse embryos, respectively (1, 2, 3, 4). They differ in developmental characteristics, transcriptomes, and signaling requirements (5). Key pathways like LIF/STAT3, BMP4, and Wnt are essential for maintaining the self-renewal and pluripotency of mouse ESCs (6, 7, 8, 9, 10, 11). In contrast, EpiSCs, derived from embryos between E5.5 and E8.0, rely on activin/nodal/transforming growth factor (TGF)-β and fibroblast growth factor (FGF) signaling pathways instead of LIF/STAT3 (2, 3, 12).

Epigenetic regulators, such as the BRG1/BRM-associated factors (BAF) complex, play crucial roles in ESC maintenance and differentiation (13). The BAF complex, an ATP-dependent chromatin-remodeling complex, is composed of 9 to 11 subunits and regulates gene expression. It is categorized into canonical BAF (cBAF), polybromo-associated BAF (PBAF), and noncanonical BAF (ncBAF) subtypes (13, 14). Inactivating specific BAF subunits leads to various abnormal phenotypes in ESCs (13). For example, deleting BRG1, the catalytic subunit, results in embryo lethality and the failure to derive ESCs from the inner cell mass (15, 16). Loss of *Dpf2* disrupts *Tbx3* regulation, impairing the mesoendoderm differentiation (17). However, the BAF complex's role and mechanisms in mouse EpiSCs are not well understood.

ESCs and EpiSCs can interconvert under certain conditions (18, 19, 20). Overexpressing ESC-specific transcription factors or activating LIF/STAT3 in EpiSCs can convert them into ESCs (18, 19, 20). Conversely, replacing LIF with FGF and activin A can convert ESCs into EpiSCs (21). However, the BAF complex's role in this transdifferentiation remains unclear.

This study shows that the LIF/STAT3 and Wnt signaling pathways, along with the cBAF and polycomb repressive complex 2 (PRC2) complexes, work together to inhibit EpiSC gene expression, preventing ESCs from converting to EpiSCs. Removing LIF reduces LIF/STAT3 signaling, lifting this inhibition and increasing TGF/nodal pathway activity. The cBAF complex then promotes EpiSC gene expression, facilitating ESC to EpiSC conversion. During this process, inhibiting PRC2 does not further upregulate EpiSC gene expression but increases STAT3 phosphorylation, which inhibits EpiSC gene expression. Unlike cBAF, inhibiting the ncBAF complex downregulates TGF-β signaling, hindering ESC to EpiSC conversion and EpiSC maintenance. In summary, this research reveals how distinct BAF complexes interact with specific signaling pathways to regulate ESC to EpiSC conversion and EpiSC maintenance.

## Results

### cBAF complex represses the differentiation of ESCs to epiblast stem cells

**Figure 1:**
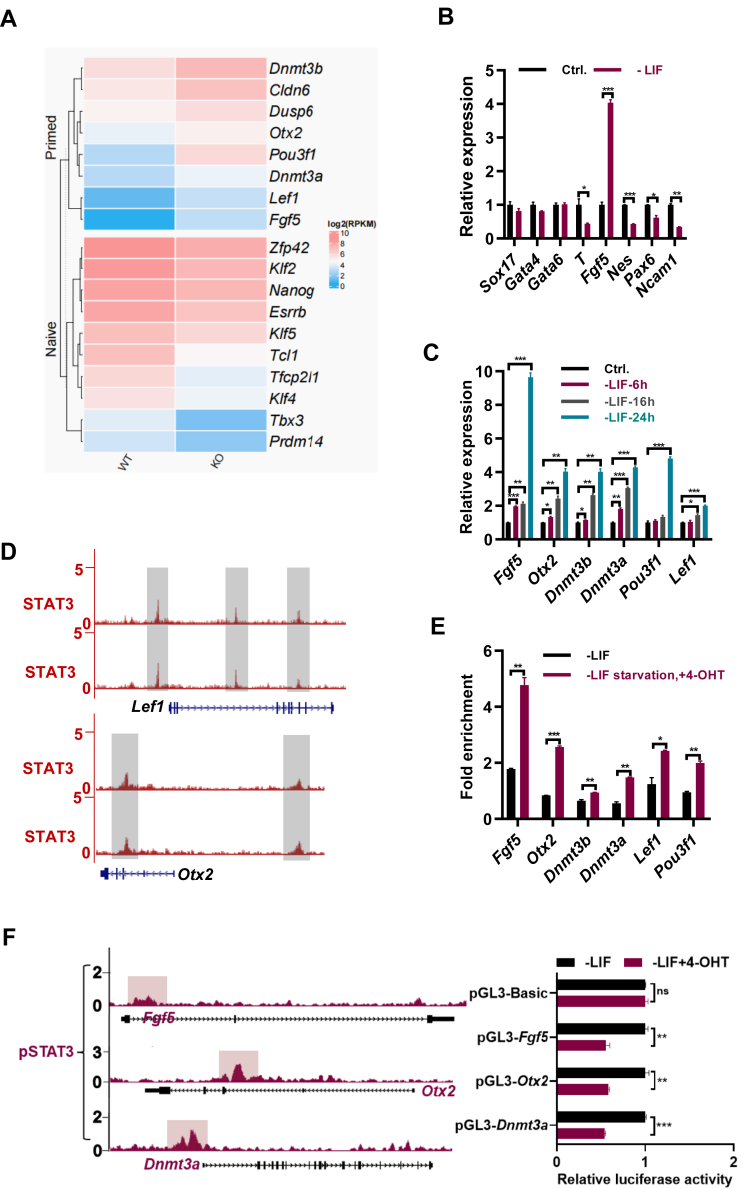
**The cBAF complex collaborates with the LIF/STAT3 pathway to repress the transition ESCs to EpiSCs.***A*, heat map depicting the expression changes of naïve and primed genes in WT and *Dpf2* KO ESCs. The color gradient represents the log2 FPKM (Fragments Per Kilobase Million) values of the genes. *B*, qPCR analysis of transcript levels for lineage marker genes in ESCs cultured for 24 h in ESC medium, with or without the addition of LIF. *C*, qPCR analysis of transcript levels for *Fgf5*, *Otx2*, *Dnmt3b*, *Dnmt3a*, *Lef1*, and *Pou3f1* genes in ESCs cultured in embryonic stem cell medium without LIF for the specified duration. *D*, genome browser view of ChIP-seq tracks for STAT3 in ESCs at the *Lef1* and *Otx2* loci. *E*, ChIP-qPCR analysis of STAT3 levels at the promoter regions of the *Fgf5*, *Otx2*, *Dnmt3b*, *Dnmt3a*, *Lef1*, and *Pou3f1* genes in ESCs cultured in ESC medium without LIF for 24 h, followed by an additional 48 h without LIF, with and without 1 μM of 4-OHT treatment. *F*, quantification of luciferase activity in STAT3-ERT2 ESCs cultured in ESC medium without LIF, with and without 1 μM of 4-OHT, under the control of the basic promoter and the promoters of the *Fgf5*, *Otx2*, and *Dnmt3a* genes. The binding regions of p-STAT3 associated with the *Fgf5*, *Otx2*, and *Dnmt3a* genes were delineated in *dark red color*, cloned into pGL3-Basic vectors, and used in luciferase assay experiments. Quantification and statistical analysis. ∗ indicates *p* < 0.05, ∗∗ indicates *p* < 0.01, ∗∗∗ indicates *p* < 0.001, ∗∗∗ indicates *p* < 0.001. Error bars represent the standard deviation. 4-OHT, 4-hydroxytamoxifen; cBAF, canonical BAF; ChIP-seq, chromatin immunoprecipitation followed by sequencing; EpiSC, epiblast stem cell; ESC, embryonic stem cell.

Dpf2 is a specific component of cBAF complex in mouse ESCs (17). To explore the role of cBAF complex on the differentiation of ESCs to EpiSCs, we analyzed the change of gene expression profile upon the deletion of *Dpf2* in mESCs. RNA-seq analysis revealed the decreased expression of pluripotency genes such as *Klf2*, *Klf4*, *Klf5*, *Tbx3*, *Nanog*, *Esrrb*, so on in *Dpf2* KO ESCs, while the expression of typical genes of EpiSCs *Dnmt3b*, *Cldn6*, *Dusp6*, *Otx2*, *Pou3f1*, *Dnmt3a*, *Lef1*, and *Fgf5* increased (Fig. 1*A*; 17). Quantitative PCR (qPCR) analysis further confirmed the increased expression of *Fgf5*, *Otx2*, *Dnmt3a*, and *Lef1* in *Dpf2* KO ESCs (Fig. S1*A*). Hence, cBAF maintains the identity of ESCs through the suppression of EpiSC gene expression.

### LIF/STAT3 suppresses the expression of EpiSC gene expression

The essential role of the LIF/STAT3 pathway in maintaining mouse ESCs has been well-documented (22). We speculated that cBAF complex may inhibit the EpiSC differentiation of ESCs with STAT3. Therefore, we explored the role of LIF/STAT3 pathway on the EpiSC differentiation of ESCs. As anticipated, the removal of LIF from LIF/serum ESC medium for 24 h led to a significant downregulation of pluripotency genes, including *Oct4*, *Sox2*, *Nanog*, *Klf4*, and *Esrrb* (Fig. S1*B*). Notably, the withdrawal of LIF for 24 h resulted in a significant upregulation of the *Fgf5* gene (Fig. 1*B*). While the expression of endoderm marker genes *Sox17*, *Gata4*, and *Gata6* showed minor changes, the expression of mesoderm gene *T* and neural ectoderm genes *Nes*, *Pax6*, and *Ncam1* decreased upon LIF withdrawal for 24 h (Fig. 1*B*).

Given that *Fgf5* is a well-established marker gene for EpiSCs (23), and LIF/STAT3 pathway is not required for the maintenance of EpiSCs (10), we hypothesized that LIF/STAT3 pathway may play a repressive role in the transition from ESCs to EpiSCs. Indeed, besides *Fgf5*, the expression of other typical EpiSC genes, including *Otx2*, *Dnmt3a*, and *Dnmt3b*, *Pou3f1, and Lef1* was significantly upregulated after 6, 16, and 24 h of LIF removal (Fig. 1*C*). Reintroduction of LIF after 24 h of LIF starvation restored the upregulated expression of *Fgf5*, *Otx2*, *Dnmt3a*, *Dnmt3b*, and *Pou3f1* in the absence of LIF (Fig. S1*C*). Hence, LIF/STAT3 may directly regulate the expression of EpiSC genes.

The administration of 4-hydroxytamoxifen (4-OHT) to STAT3-ERT2-expressing cells resulted in the translocation of STAT3-ERT2 into the nucleus and subsequent activation of STAT3 targets (24, 25, 26). Therefore, we generated STAT3-ERT2 ESCs (Fig. S1*D*). As expected, the addition of 4-OHT in the absence of LIF increased the protein levels of both STAT3 and p-STAT3 (Fig. S1*E*), indicating the increased activity of LIF/STAT3 pathway. Consistently, the addition of 4-OHT upregulated the expression of the target genes of the LIF/STAT3 pathway, including *Nanog*, *Tbx3*, *Mras*, *Eya1*, *Stat3*, *Gjb3*, *Lama1*, *Esrrb*, *Fabp3*, and *Ppap2b* (Fig. S1*F*), demonstrating the activation of the LIF/STAT3 pathway. Furthermore, the addition of 4-OHT restored the upregulated expression of *Fgf5*, *Otx2*, *Dnmt3a*, *Dnmt3b*, *Lef1*, and *Pou3f1* genes upon the withdrawal of LIF (Fig. S1*G*), further supporting the repressive role of LIF/STAT3 pathway on the expression of EpiSC genes.

To explore the direct regulation of EpiSC genes by STAT3, chromatin immunoprecipitation followed by sequencing (ChIP-seq) analysis with a STAT3 antibody was analyzed (17). The enrichment of STAT3 at the promoter regions of EpiSC genes such as *Otx2* and *Lef1* was evident (Fig. 1*D*). The withdrawal of LIF resulted in the decreased STAT3 binding at the *Fgf5*, *Otx2*, *Dnmt3a*, Dnmt3b, *Lef1*, and *Pou3f1* genes, as confirmed by ChIP-qPCR experiments (Fig. S1*H*). Conversely, the enrichment of STAT3 at *Fgf5*, *Otx2*, *Dnmt3a*, *Dnmt3b*, *Lef1*, and *Pou3f1* increased upon the addition of 4-OHT to STAT3-ERT2-expressing cells cultured in the absence of LIF (Fig. 1*E*), providing further evidence that STAT3 directly represses their expression of EpiSC genes.

To further validate the direct regulation of STAT3 on *Fgf5*, *Otx2*, and *Dnmt3b* genes, luciferase assays were conducted. Luciferase vectors were constructed by cloning the promoter regions of *Fgf5*, *Otx2*, and *Dnmt3a* genes, which encompassed the STAT3 binding sites, into the pGL3-basic vector (Fig. 1*F*). Subsequently, these vectors were transfected into STAT3 ERT2-expressing ESCs with and without the addition of 4-OHT in the absence of LIF. The luciferase assays revealed the reduced promoter activities of *Fgf5*, *Otx2*, and *Dnmt3a* in STAT3 ERT2-expressing cells upon the addition of 4-OHT in the absence of LIF (Fig. 1*F*), demonstrating the repressive role of STAT3 on the promoter activities of the EpiSC genes. As expected, the expression of *Fgf5*, *Otx2*, *Dnmt3a*, *Dnmt3b*, *Lef1*, and *Pou3f1* genes was significantly increased in N2B27 medium supplemented with activin A and basic fibroblast growth factor (bFGF), which was repressed upon the addition of LIF (Fig. S1*I*). In summary, the LIF/STAT3 pathway directly inhibits the expression of EpiSC genes such as *Fgf5*, *Otx2*, and *Dnmt3a* in ESCs and during the differentiation of ESCs to EpiSCs.

BAF complex in ESCs has been reported to stabilize the binding of STAT3 to the genome, thereby facilitating the maintenance of ESCs (27). Considering the repressive role of Dpf2 on EpiSC gene expression, we speculated that Dpf2 may repress the expression of EpiSC genes by maintaining the activity of LIF/STAT3 signaling. Indeed, the deletion of *Dpf2* resulted in the downregulation of LIF/STAT3 target genes, including *Mras*, *Ly6g6e*, *Cobl*, *Gjb3*, *Lrrc34*, *Fabp3*, and *Lama1* (Fig. S1*J*), supporting a notion that Dpf2 regulates EpiSC gene expression through the modulation of LIF/STAT3 pathway activity.

### LIF/STAT3 pathway collaborates with PRC2 complex to repress the expression of EpiSC genes in ESCs

**Figure 2:**
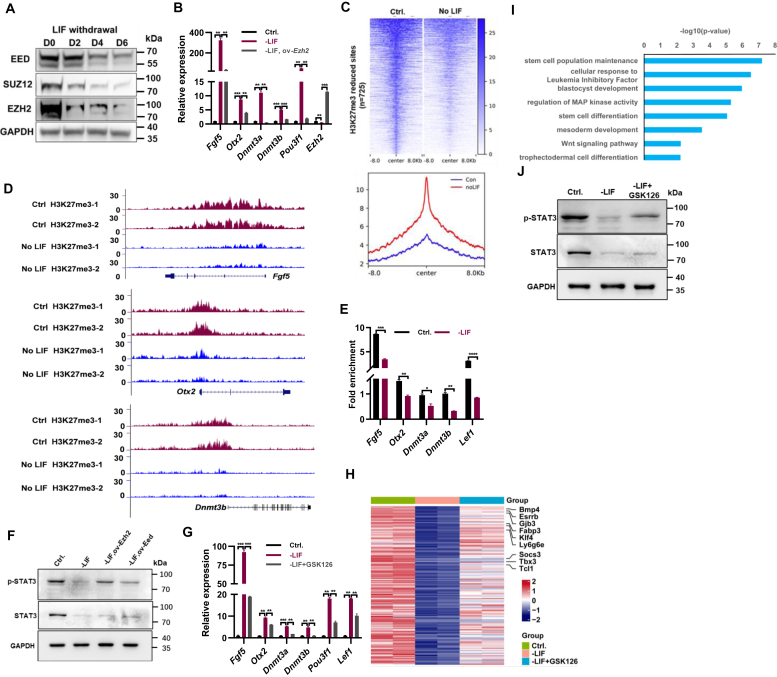
**cBAF complex and LIF/STAT3 pathway collaboratively repress the transition of ESCs to EpiSCs.***A*, Western blot analysis depicting protein levels of EED, SUZ12, and EZH2 in ESCs cultured in ESC medium without LIF for the specified duration. GAPDH was used as a loading control. *B*, qPCR analysis of transcript levels for *Fgf5*, *Otx2*, *Dnmt3a*, *Dnmt3b*, and *Pou3f1* in ESCs cultured in ESC medium in the absence of LIF, with and without *Ezh2* overexpression for 48 h. *C*, heatmap representation of normalized tag density profiles depicting reduced H3K27me3 in ESCs cultured in ESC medium with and without LIF, along with corresponding metaplots illustrating signal intensities. *D*, genome browser view of ChIP-seq tracks for H3K27me3 at the *Fgf5*, *Otx2* and *Dnmt3b* loci in ESCs cultured with (Ctrl.) and without LIF (-LIF). *E*, ChIP-qPCR analysis of H3K27me3 levels at the promoter regions of the *Fgf5*, *Otx2*, *Dnmt3a*, *Dnmt3b*, and *Lef1* genes in ESCs cultured in ESC medium with and without LIF for 48 h. *F*, Western blot analysis depicting protein levels of STAT3, and p-STAT3 in ESCs cultured in ESC medium without LIF, with and without overexpression of *Ezh2* or *Eed* for 48 h. GAPDH was used as a loading control. *G*, qPCR analysis of transcript levels for *Fgf5, Otx2*, *Dnmt3a*, *Dnmt3b*, *Lef1* and *Pou3f1* in ESCs cultured in ESC medium in the absence of LIF, with and without 10 μM of GSK126. *H*, heatmap illustrating downregulated genes in the absence of LIF that are upregulated upon 10 μM of GSK126 treatment, identified through RNA-seq analysis in ESCs cultured in standard ESC medium (Ctrl.), ESC medium without LIF (-LIF), and ESC medium without LIF with 10 μM of GSK126. *I*, GO analysis for biological processes associated with genes differentially expressed identified in (*H*). *J*, Western blot analysis depicting protein levels of STAT3, and p-STAT3 in ESCs cultured in ESC medium without LIF, with and without 10 μM of GSK126 for 48 h. GAPDH was used as a loading control. ∗ indicates *p* < 0.05, ∗∗ indicates *p* < 0.01, ∗∗∗ indicates *p* < 0.001, ∗∗∗∗ indicates *p* < 0.001. Error bars represent the standard deviation. cBAF, canonical BAF; ChIP-seq, chromatin immunoprecipitation followed by sequencing; EpiSC, epiblast stem cell; ESC, embryonic stem cell; GO, Gene Ontology.

The PRC2 proteins are conserved chromatin modification factors best known for silencing gene expression by regulating chromatin through H3K27me3 (28). We speculated that STAT3 may collaborate with PRC2 to repress the expression of EpiSC genes in ESCs. Both the mRNA and protein levels of PRC2 subunits *Ezh2*, *Suz12*, and *Eed* decreased in ESCs cultured in non-LIF medium, which was restored upon the addition of LIF (Figs. 2*A*; S2*A*). Therefore, the inactivation of LIF/STAT3 pathway may result in the derepression of EpiSC genes by reducing the activity of PRC2 complex. Consistently, overexpression of either *Ezh2*, or *Eed* restored the upregulated expression of *Fgf5*, *Otx2*, *Dnmt3a*, *Dnmt3b*, and *Pou3f1* upon the withdrawal of LIF (Figs. 2*B*; S2*B*). Further, we carried out ChIP-seq experiment with H3K27me3 antibody with ESCs cultured in the medium supplemented with and without LIF. ChIP-seq analysis identified 725 sites with significant reduction (≥2-fold) of H3K27me3 in ESCs cultured in the absence of LIF (Fig. 2*C*). We also observed 1044 sites that gained H3K27me3 signal by 2-fold or more in ESCs cultured in the absence of LIF (Fig. S2*C*). Both H3K27me3 reduced and gained sites were located in the vicinity of genes associated with cell fate commitment, brain and heart development, so on (Fig. S2*D*).

The esBAF complexes, the BAF complex expressed in ESCs, act synergistically with STAT3 to maintain the expression of LIF/STAT3 target genes in ESCs by antagonizing PRC2 complex (27). As expected, the withdrawal of LIF resulted in the accumulated H3K27me3 deposition at LIF/STAT3 target genes, such as *Tbx3*, *Gjb3*, and *Mras* (Fig. S2*E*). In contrast, the H3K27me3 modification decreased at EpiSC genes *Fgf5*, *Otx2*, and *Dnmt3b* upon the depletion of LIF in ESC medium (Fig. 2*D*). ChIP-qPCR experiments further confirmed the decreased H3K27me3 at EpiSC genes *Fgf5*, *Otx2*, *Dnmt3a*, *Dnmt3b*, and *Lef1* (Fig. 2*E*). In summary, distinct to the antagonizing role of STAT3 and PRC2 on LIF/STAT3 target gene expression, STAT3 and PRC2 complex collaboratively repress the EpiSC gene expression in ESCs.

Overexpressing either *Ezh2* or *Eed* restored the upregulated EpiSC gene expression induced by the depletion of LIF in ESCs (Figs. 2*B*; S2*B*). EZH2 activates STAT3 signaling *via* STAT3 methylation in leukemia cells (29). Therefore, we speculated that overexpression of *Ezh2* or *Eed* may increase the activity of STAT3 signaling, thereby repress the expression of EpiSC genes in ESCs. Indeed, overexpressing either *Ezh2* or *Eed* partially restored the diminished p-STAT3 level upon LIF depletion (Fig. 2*F*). In consistent, overexpression of either *Ezh2* or *Eed* partially restored the reduced expression of LIF/STAT3 target genes *Stat3*, *Ly6g6e*, *Esrrb*, *Mras*, and *Tbx3* in ESCs cultured in the absence of LIF (Fig. S2, *F* and *G*). Therefore, overexpression of PRC2 components Eed, or Ezh2 increased the activation of STAT3, thereby repressed the upregulated EpiSC gene expression upon LIF deprivation.

Contrary to the anticipated inhibition of EpiSC gene expression by overexpression of *Ezh2* or *Eed* in the absence of LIF, inhibition of EZH2 using its inhibitor GSK126 also suppressed the expression of EpiSC-specific genes including *Fgf5*, *Otx2*, *Dnmt3a*, *Dnmt3b*, *Pou3f1*, and *Lef1*, which were otherwise induced following LIF withdrawal (Figs. 2, *B* and *G*; S2*B*). To elucidate the underlying mechanisms, RNA-seq experiment was performed on ESCs cultured without LIF, both with and without GSK126 treatment. Analysis of the RNA-seq data revealed that 219 genes, including EpiSC markers *Fgf5*, *Dnmt3a*, *Dnmt3b*, and *Pou3f1*, were upregulated in the absence of LIF, but exhibited reduced expression following GSK126 treatment (Fig. S2*H*; Table S2), corroborating the qPCR findings (Fig. 2*G*). Gene Ontology (GO) analysis associated these genes with the regulation of neurogenesis, cardiac rate, vascular and muscle cell differentiation, and DNA methylation-dependent heterochromatin assembly (Fig. S2*I*). Conversely, 150 genes downregulated in the absence of LIF were upregulated upon GSK126 treatment, relating to stem cell population maintenance, cellular responses to LIF, Wnt signaling pathway, and stem cell differentiation among other processes (Fig. 2, *H* and *I*; Table S2). Notably, genes upregulated by GSK126 treatment, including LIF/STAT3 pathway targets such as *Bmp4*, *Esrrb*, *Gjb3*, *Fabp3*, *Klf4*, *Ly6g6e*, *Socs3*, *Tbx3*, and *Tcl1*, suggest an enhancement of STAT3 signaling upon EZH2 inhibition under LIF-deficient conditions (Fig. 2*H*). This observation was further supported by elevated protein levels of STAT3 and p-STAT3 (Fig. 2*J*). Additionally, qPCR analysis confirmed that the diminished expression of LIF/STAT3 target genes *Tbx3*, *Mras*, *Eya1*, *Ly6g6e*, *Esrrb*, *Stat3*, *Gjb3*, and *Fabp3* in the absence of LIF was restored with GSK126 treatment (Fig. S2*J*). Therefore, the inhibition of PRC2 with GSK126 represses the EpiSC gene expression *via* upregulating the activity of LIF/STAT3 pathway. In summary, both the overexpression of Ezh2 and the inhibition of Ezh2 by GSK126 led to an increase in LIF/STAT3 activity, thereby repressing EpiSC gene expression.

### LIF/STAT3 pathway inhibits the expression of EpiSC genes *via* maintaining the activity of Wnt pathway

**Figure 3:**
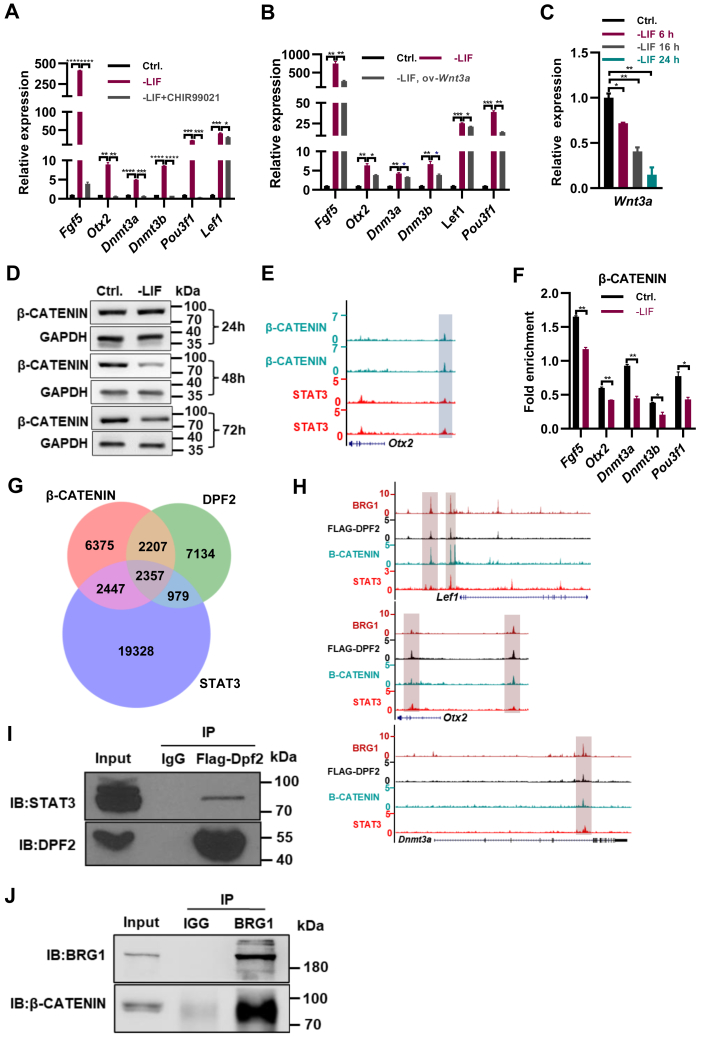
**LIF/STAT3 pathway inhibits the expression of EpiSC genes *via* maintaining the activity of Wnt pathway.***A*, qPCR analysis of transcript levels for *Fgf5*, *Otx2*, *Dnmt3a*, *Dnmt3b*, *Pou3f1*, and *Lef1* in ESCs cultured in ESC medium in the absence of LIF, with and without 3 μM of CHIR99021 activator for 48 h. *B*, qPCR analysis of transcript levels for *Fgf5*, *Otx2*, *Dnmt3a*, *Dnmt3b*, *Pou3f1*, and *Lef1* in ESCs cultured in ESC medium in the absence of LIF, with and without overexpression of *Wnt3a* for 48 h. *C*, qPCR analysis of transcript levels for *Wnt3a* in ESCs cultured in ESC medium without LIF for the specified duration. *D*, Western blot analysis depicting protein levels of β-CATENIN in ESCs cultured in ESC medium without LIF for the specified duration. GAPDH was used as a loading control. *E*, genome browser view of ChIP-seq tracks for β-CATENIN and STAT3 at the *Otx2* loci in ESCs. *F*, ChIP-qPCR analysis of β-CATENIN levels at the promoter regions of the *Fgf5*, *Otx2*, *Dnmt3a*, *Dnmt3b*, and *Pou3f1* genes in ESCs cultured in ESC medium with and without LIF for 48 h. *G*, Venn diagram depicting the number of genes that are targeted by DPF2, β-CATENIN, and STAT3 from DPF2, β-CATENIN, and STAT3 ChIP-seq analyses. *H*, genome browser view of ChIP-seq tracks for BRG1, DPF2, β-CATENIN, and STAT3 at the *Lef1*, *Otx2*, and *Dnmt3a* loci in ESCs. *I*, confirmation of the interaction between FLAG-DPF2 and STAT3 in ESCs through Co-IP followed by Western blot analysis. *J*, confirmation of the interaction between BRG1 and β-CATENIN in ESCs through Co-IP followed by Western blot analysis. ∗ indicates *p* < 0.05, ∗∗ indicates *p* < 0.01, ∗∗∗ indicates *p* < 0.001, ∗∗∗∗ indicates *p* < 0.001. Error bars represent the standard deviation. ChIP-seq, chromatin immunoprecipitation followed by sequencing; Co-IP, co-immunoprecipitation; EpiSC, epiblast stem cell; ESC, embryonic stem cell; qPCR, quantitative PCR.

Both paracrine and autocrine Wnt signals are essential for ESC self-renewal and are required to inhibit their differentiation into EpiSCs (30, 31, 32). Consistent with this, the activation of the Wnt pathway with 3 μM of CHIR99021 activator repressed the expression of *Fgf5*, *Otx2*, *Dnmt3a*, *Dnmt3b*, *Pou3f1*, and *Lef1* in ESCs cultured in ESC medium with or without the addition of LIF (Figs. 3*A*; S3*A*). The withdrawal of CHIR99021 in 2i medium also resulted in the elevated expression of *Fgf5*, *Otx2*, *Dnmt3a*, *Dnmt3b*, and *Pou3f1* (Fig. S3*B*). Overexpression of *Wnt3a* restored the upregulated expression of *Fgf5*, *Otx2*, *Dnmt3a*, *Dnmt3b*, *Lef1*, and *Pou3f1* resulted from the withdrawal of LIF (Fig. 3*B*). In 2i/LIF medium, the withdrawal of either LIF, or CHIR99021 resulted in the upregulation of EpiSC genes, while the simultaneous withdrawal of LIF and CHIR99021 led to more significant elevation of *Fgf5*, *Otx2*, *Dnmt3a*, *Dnmt3b*, *Lef1*, and *Pou3f1* genes (Fig. S3*C*). Therefore, LIF/STAT3 and Wnt pathways collaboratively repress the EpiSC gene expression in ESCs. In line with that, the withdrawal of LIF induced a shift in the morphological characteristics of ESCs, leading them to closely resemble the morphology observed in EpiSCs (Fig. S3*D*). This alteration in ESC morphology was subsequently restored upon the administration of the CHIR99021 activator (Fig. S3*D*). Interestingly, the withdrawal of LIF downregulated the transcript levels of *Wnt3a* and *Ctnnb1*, the gene coding for β-CATENIN, and the protein levels of β-CATENIN (Figs. 3, *C* and *D*; S3*E*). The addition of 4-OHT in STAT3 ERT2-expressing cells restored the downregulated *Wnt3a* expression upon the depletion of LIF (Fig. S3*F*), indicating the direct regulation of Wnt3a by LIF/STAT3 pathway. ChIP-seq analysis revealed the increased H3K27me3 at *Wnt3a* gene in ESCs cultured in ESC medium without LIF (Fig. S3*G*), further confirmed by ChIP-qPCR (Fig. S3*H*). Therefore, besides the direct repression of STAT3 on EpiSC gene expression, LIF/STAT3 pathway represses the expression of EpiSC genes *via* maintaining the activity of Wnt pathway. ChIP-seq analysis revealed the cobinding of β-CATENIN and STAT3 at the *Otx2*, *Dnmt3a*, and *Lef1* loci (Figs. 3*E*; S3*I*). The binding of β-CATENIN at the *Fgf5*, *Otx2*, *Dnmt3a*, *Dnmt3b*, and *Pou3f1* loci was reduced upon the withdrawal of LIF (Fig. 3*F*), supporting the collaborative inhibition of LIF/STAT3 and Wnt pathways on the differentiation of ESCs into EpiSCs.

### cBAF complex collaborates with LIF/STAT3, Wnt pathways to repress the transition of ESCs to EpiSCs

The cBAF complex has been reported to facilitate pluripotency by stabilizing the binding of STAT3 to its target genes (27). Inhibition of Dpf2, the LIF/STAT3 pathway, or the Wnt pathway led to the upregulation of EpiSC-associated genes (Figs. 1 and 2). Consequently, we investigated the collaborative regulation of cBAF with the LIF/STAT3 and Wnt pathways on the differentiation of ESCs to EpiSCs. ChIP-seq analysis for DPF2, β-CATENIN, and STAT3 identified 2357 peaks cobound by DPF2, β-CATENIN, and STAT3, associated with 1880 genes, including EpiSC genes *Fgf5*, *Lef1*, *Otx2*, and *Dnmt3a* (Fig. 3*G*; Table S2), and specifically at the *Lef1*, *Otx2*, and *Dnmt3a* loci (Fig. 3*H*). GO analysis of the common target genes of DPF2, β-CATENIN, and STAT3 revealed functions related to pluripotency, TGF-β signaling, and chromatin organization (Fig. S3*J*). Coimmunoprecipitation experiments confirmed interactions between the BAF complex components DPF2 and BRG1 with STAT3 and β-CATENIN (Fig. 3, *I* and *J*). In summary, the LIF/STAT3 and Wnt pathways, along with the cBAF complex, collaboratively maintain ESC self-renewal by repressing the transition of ESCs to EpiSCs.

### BAF complex is required to maintain the expression of EpiSC genes

**Figure 4:**
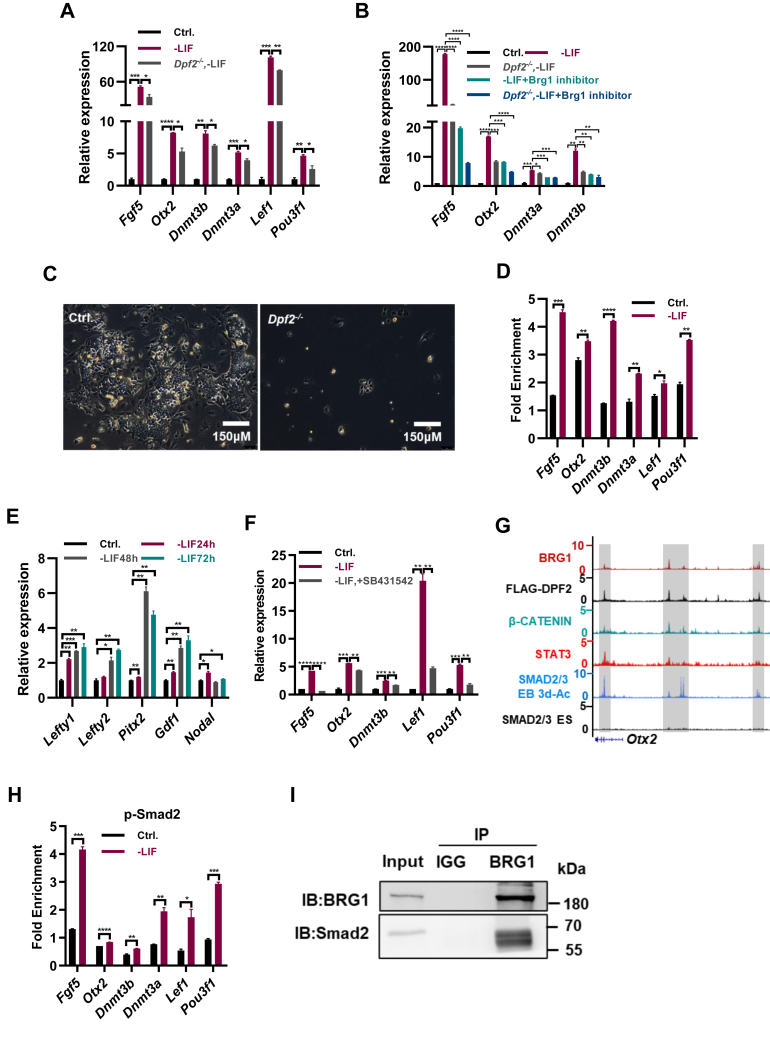
**The cBAF complex collaborates with the TGF-β pathway to maintain the expression of EpiSC genes.***A*, qPCR analysis of *Fgf5*, *Otx2*, *Dnmt3a*, *Dnmt3b*, *Pou3f1*, and *Lef1* transcript levels in ESCs cultured in standard ESC medium (Ctrl.), ESC medium without LIF (-LIF), and *Dpf2* KO ESCs cultured in ESC medium without LIF for 48 h. *B*, qPCR analysis of *Fgf5*, *Otx2*, *Dnmt3a*, and *Dnmt3b* transcript levels in WT and *Dpf2* KO ESCs cultured in the indicated conditions. *C*, morphology of WT and *Dpf2* KO EpiSCs cultured and in AF medium passaged twice. *D*, ChIP-qPCR analysis of BRG1 levels at the promoter regions of the *Fgf5*, *Otx2*, *Dnmt3a*, *Dnmt3b*, *Lef1*, and *Pou3f1* genes in ESCs cultured in ESC medium with and without LIF for 48 h. *E*, qPCR analysis of *Lefty1*, *Lefty2*, *Pitx2*, *Gdf1*, and *Nodal* transcript levels in ESCs cultured without LIF for the specified duration. *F*, qPCR analysis of *Fgf5*, *Otx2*, *Dnmt3b*, *Lef1*, and *Pou3f1* transcript levels in ESCs cultured in the absence of LIF for 48 h with and without 10 μM of SB431542. *G*, genome browser view of ChIP-seq tracks depicting BRG1, DPF2, β-CATENIN, and STAT3 binding in WT ESCs, SMAD2/3 binding in WT ESCs, and day 3 EBs induced with activin A (ACT) at the *Otx2* locus. *H*, ChIP-qPCR analysis of SMAD2 levels at the promoter regions of the *Fgf5*, *Otx2*, *Dnmt3b*, *Dnmt3a*, *Lef1*, and *Pou3f1* genes in ESCs cultured in ESC medium with and without LIF for 48 h. *I*, confirmation of the interaction between BRG1 and SMAD2 in ESCs through Co-IP followed by Western blot analysis. The Western blot for BRG1 was identical to that shown in Figure 3*J*, as both panels were obtained from the same Co-IP experiment. ∗ indicates *p* < 0.05, ∗∗ indicates *p* < 0.01, ∗∗∗ indicates *p* < 0.001, ∗∗∗∗ indicates *p* < 0.001. Error bars represent the standard deviation. cBAF, canonical BAF; ChIP-seq, chromatin immunoprecipitation followed by sequencing; Co-IP, co-immunoprecipitation; EpiSC, epiblast stem cell; ESC, embryonic stem cell; qPCR, quantitative PCR; TGF, transforming growth factor.

The inactivation of LIF/STAT3 signaling or the deletion of Dpf2 upregulated the expression of EpiSC-associated genes such as *Fgf5*, *Otx2*, *Dnmt3a*, and *Dnmt3b* (Figs. 1, *A* and *C*; S1, *A* and *C*). We hypothesized that inactivation of the cBAF complex could lead to further increased expression levels of EpiSC genes following LIF withdrawal. Contrary to expectations, the deletion of *Dpf2* did not enhance the expression of *Fgf5*, *Otx2*, *Dnmt3a*, *Dnmt3b*, *Lef1*, and *Pou3f1* in ESCs cultured without LIF; instead, it resulted in downregulation of these genes (Fig. 4*A*). BRG1, a core subunit of the BAF complex in ESCs, when inhibited by the BRM/BRG1 ATP Inhibitor-1, restored the LIF withdrawal-induced elevated expression of *Fgf5*, *Otx2*, *Dnmt3a*, and *Dnmt3b* (Fig. 4*B*). Moreover, simultaneous inhibition of both BRG1 and DPF2 further decreased the upregulated expression of these genes (Fig. 4*B*).

To further elucidate Dpf2's role in sustaining EpiSCs, *Dpf2*^*fl/fl*^ EpiSCs were derived from *Dpf2*^*fl/fl*^ ESCs using AF medium (serum-free medium containing activin A and bFGF). Deletion of *Dpf2* through 4-OHT treatment resulted in EpiSC death (Fig. 4*C*). Similarly, treatment with BRM/BRG1 ATP Inhibitor-1 led to failure in EpiSC maintenance (Fig. S4*A*). These findings suggest that the BAF complex is crucial for maintaining the transcriptional activity of EpiSC-specific genes following LIF withdrawal.

BRG1 is known to counteract PRC2 to establish chromatin accessibility at STAT3 binding sites (27). Consistent with this, LIF withdrawal reduced BRG1 binding at LIF/STAT3 target genes such as *Gjb3*, *Eya1*, *Mras*, and *Lama1* (Fig. S4*B*). In contrast, BRG1 binding at *Fgf5*, *Otx2*, *Dnmt3a*, *Dnmt3b*, *Lef1*, and *Pou3f1* increased in ESCs cultured without LIF (Fig. 4*D*), supporting the role of the BAF complex in maintaining EpiSC gene expression. Additionally, *Dpf2* KO restored the reduced H3K27me3 deposition at EpiSC genes following LIF withdrawal (Fig. S4*C*), indicating that DPF2 is necessary for maintaining low levels of repressive histone modifications at EpiSC gene loci. Thus, the cBAF complex maintains EpiSC gene expression in the absence of LIF by antagonizing PRC2.

### cBAF complex collaborates with TGF-**β** pathway to maintain the expression of EpiSC genes

The cBAF complex is imperative for sustaining the expression of EpiSC genes following LIF withdrawal (Fig. 4, *A* and *B*). We hypothesized that alternative signaling pathway(s)/transcription factor(s) may substitute for LIF/STAT3 pathway/STAT3 binding, thereby synergizing with the cBAF complex to uphold EpiSC gene expression, thereby facilitating the transition of ESCs to EpiSCs. Activin/nodal/TGF-β signaling exhibits a conserved role in EpiSC derivation and maintenance (2, 3). The expression of genes related to the TGF-β pathway, including *Lefty1*, *Lefty2*, *Pix2*, *Gdf1*, and *Nodal*, increased in ESCs cultured without LIF or in *Dpf2* KO ESCs (Figs. 4*E*; S4*D*), indicating enhanced activin/nodal/TGF-β signaling activity. This heightened activity potentially contributes to the increased expression of EpiSC genes in ESCs cultured without LIF or in *Dpf2* KO ESCs (Figs. 1*A*; S1*A*). Indeed, the inhibition of the TGF-β pathway with SB431542 downregulated the heightened expression of *Fgf5*, *Otx2*, *Dnmt3b*, *Lef1*, and *Pou3f1* induced by LIF withdrawal (Fig. 4*F*). ChIP-Seq analysis for STAT3, SMAD2/3, DPF2, and BRG1 revealed their colocalization at *Otx2*, *Lef1*, and *Dnmt3a* (Figs. 4*G*; Fig. S4, *E* and *F*). Addition of activin resulted in increased SMAD2/3 binding at *Otx2*, *Lef1*, and *Dnmt3a* genes (Figs. 4*G*; S4, *E* and *F*). ChIP-qPCR demonstrated the heightened SMAD2/3 binding at *Fgf5*, *Otx2*, *Dnmt3b*, *Dnmt3a*, *Lef1*, and *Pou3f1* genes in ESCs cultured without LIF (Fig. 4*H*), which was restored upon *Dpf2* deletion (Fig. S4*G*). BRG1 inhibition reduced the expression of EpiSC genes *Fgf5*, *Otx2*, *Dnmt3a*, and *Dnmt3b* (Fig. 4*B*). Coimmunoprecipitation confirmed interaction between BRG1 and the TGF-β pathway effector protein SMAD2 (Fig. 4*I*). In conclusion, our findings suggest that the cBAF complex and TGF-β pathway cooperate to maintain expression of EpiSC genes in ESCs cultured in the absence of LIF.

### ncBAF complex maintains the ESC identity *via* repressing the TGF-**β** activity

**Figure 5:**
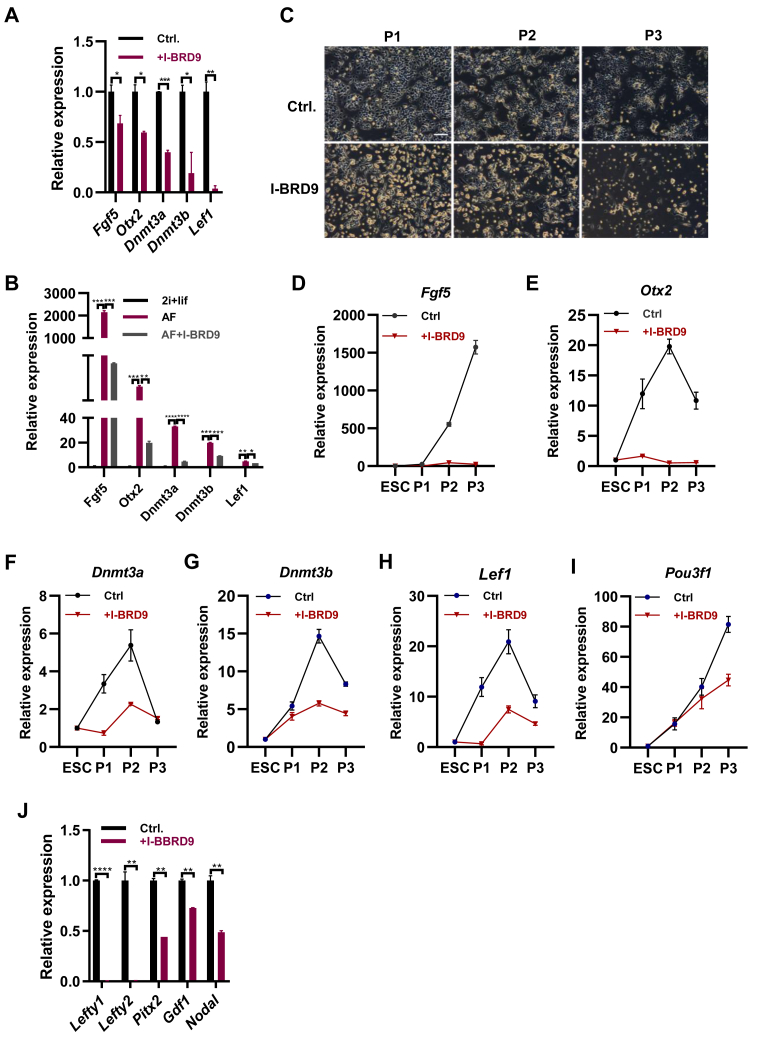
**ncBAF complex represses the transition of ESCs to EpiSCs.***A*, qPCR analysis of transcript levels for *Fgf5*, *Otx2*, *Dnmt3a*, *Dnmt3b*, and *Lef1* in ESCs cultured in ESC medium with and without the addition of 10 μM of I-BRD9. *B*, qPCR analysis of transcript levels for *Fgf5*, *Otx2*, *Dnmt3a*, *Dnmt3b*, and *Lef1* in ESCs cultured in 2i/LIF ESC medium, AF medium with and without the addition of 10 μM of I-BRD9. *C*, morphology of ESCs cultured in AF medium and passaged 1 to 3 times with 10 μM of I-BRD9 treatment. The scale bar represents 150 μm. *D*–*I*, qPCR analysis of transcript levels for *Fgf5*, *Otx2*, *Dnmt3a*, *Dnmt3b*, *Lef1*, and *Pou3f1* in ESCs cultured in ESC medium, ESCs cultured and passaged 1 to 3 times in AF medium with and without the addition of 10 μM of I-BRD9. *J*, qPCR analysis of transcript levels for *Lefty1*, *Lefty2*, *Pitx2*, *Gdf1*, and *Nodal* in ESCs cultured in ESC medium with and without 10 μM of I-BRD9. ∗ indicates *p* < 0.05, ∗∗ indicates *p* < 0.01, ∗∗∗ indicates *p* < 0.001, ∗∗∗∗ indicates *p* < 0.001. Error bars represent the standard deviation. ESC, embryonic stem cell; EpiSC, epiblast stem cell; ncBAF, noncanonical BAF; EpiSC, epiblast stem cell; qPCR, quantitative PCR.

BRD9 is a specific component of ncBAF complex (14, 33). Gatchalian *et al.* reported that ncBAF complexes have a functionally specific role in preserving the naive pluripotency of ESCs (33). Unexpectedly, the addition of BRD9 inhibitor, iBRD9 reduced the expression of *Fgf5*, *Otx2*, *Dnmt3a*, *Dnmt3b*, and *Lef1* in both LIF/Serum embryonic stem cell medium and 2i medium with bFGF4 and activin A (Fig. 5, *A* and *B*), indicating the positive role of BRD9 on the expression of EpiSC genes. In consistent, the inhibition of BRD9 also restored the upregulated expression of *Fgf5*, *Otx2*, *Dnmt3a*, D*nmt3b*, *Lef1*, and *Pou3f1* upon the withdrawal of LIF (Fig. S5*A*). During the differentiation of ESCs to EpiSCs, the addition of I-BRD9 resulted in the significant cell death (Fig. 5*C*), indicating the inhibited differentiation by I-BRD9. qPCR analysis revealed the significant downregulation of *Fgf5*, *Otx2*, *Dnmt3a*, *Dnmt3b*, *Lef1*, and *Pou3f1* upon the addition of I-BRD9 (Fig. 5, *D*–*I*). Therefore, BRD9 plays a positive role on the differentiation of ESCs to EpiSCs.

Similarly to Dpf2, the inhibition of Brd9 reduced both the protein and transcript level of Stat3 (Fig. S5, *B* and *C*), which led to the decreased expression of LIF/STAT3 target genes *Gjb3*, *Mras*, *Eya1*, *Stat3*, *Lama1*, *Ly6g6e*, and *Fabp3* (Fig. S5*C*). As the inhibition of BRD9 impaired the activity of LIF/STAT3 pathway and the LIF/STAT3 pathway inhibits the expression EpiSC genes in ESCs (Fig. 1*C*), the downregulation of EpiSC genes upon the inhibition of BRD9 may be *via* other mechanism. The inhibition of Brd9 impairs the activity of TGF-β pathway in both hESCs and cancer cells (34). We speculated that Brd9 may regulate the expression of mouse EpiSC genes *via* controlling the activity of TGF-β activity. Indeed, the inhibition of Brd9 led to the decreased activity of TGF-β pathway in mouse ESCs, revealing by the decreased protein level of SMAD2 (Fig. S5*D*), and the downregulated expression of TGF-β related genes *Lefty1*, *Lefty2*, *Pitx2*, *Gdf1*, and *Nodal* (Fig. 5*J*). Similar to the inhibition of Dpf2 and BRG1 (Figs. 4*C*; S4*A*), the inhibition of BRD9 impaired the maintenance of EpiSCs (Fig. S5*E*). Therefore, ncBAF may regulate the EpiSC differentiation of ESCs *via* controlling the activity of TGF-β pathway.

## Discussion

**Figure 6:**
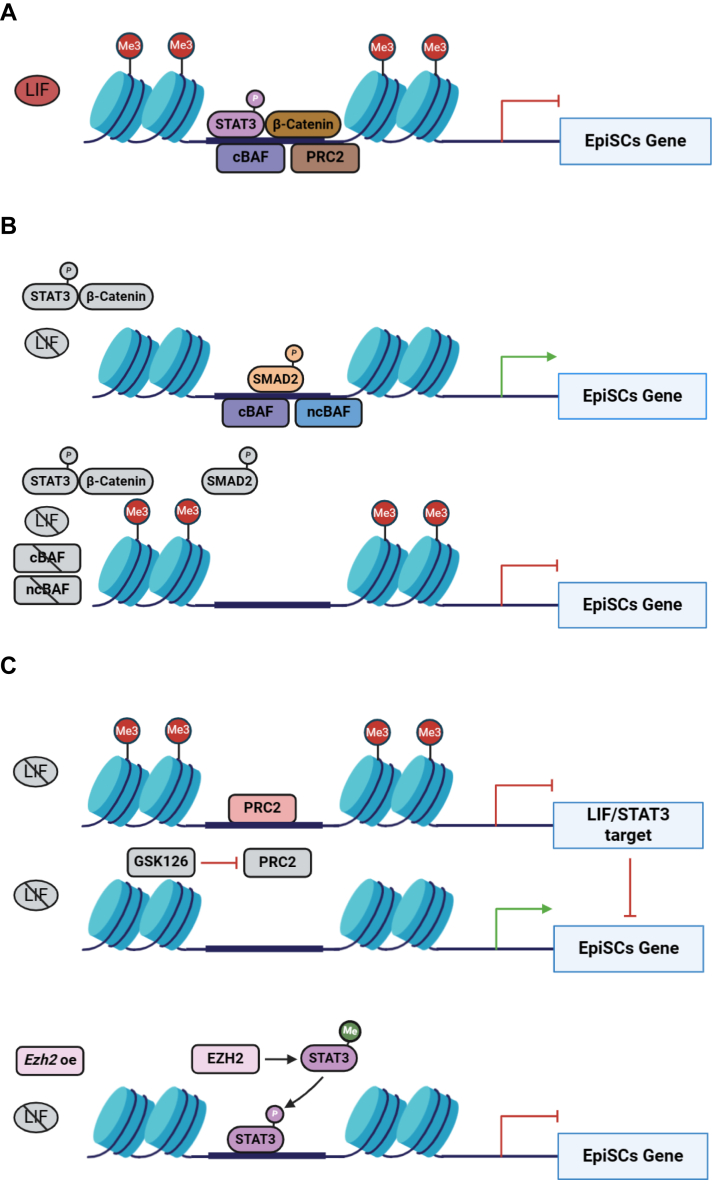
**Model for the interplay of distinct BAF complexes in embryonic and epiblast stem cell conversion and maintenance.***A*, the LIF/STAT3 and Wnt/β-Catenin signaling pathways, along with the cBAF and PRC2 complexes, cooperatively suppress the expression of EpiSCs genes. *B*, the TGF-β signaling pathway, in cooperation with the cBAF and ncBAF complexes, maintains the expression of EpiSC genes. *C*, the PRC2 complex regulates the expression of EpiSC genes through two mechanisms. cBAF, canonical BAF; EpiSC, epiblast stem cell; ncBAF, noncanonical BAF; PRC2, polycomb repressive complex 2; TGF, transforming growth factor.

The maintenance of pluripotency in mouse ESCs necessitates precise regulation by a complex network that includes pluripotency transcription factors, signaling pathways, and chromatin remodeling complexes (13, 35, 36). Major signaling pathways such as LIF/STAT3, Wnt, and TGF-β are reported to play essential roles in maintaining pluripotent ESCs and EpiSCs (37, 38). BAF chromatin remodeling complexes are crucial for the self-renewal and differentiation of ESCs in conjunction with pluripotency transcription factors and signaling pathways (13, 33, 34). However, the mechanisms by which BAF complexes collaborate with key pathways to regulate the maintenance and the conversion of EpiSCs with ESCs are not reported. In this study, we reveal that under conditions that support ESC self-renewal with LIF, the cBAF complex works with the LIF/STAT3 and WNT signaling pathways, as well as the PRC2 complex, to directly repress EpiSC genes (Fig. 6*A*). Additionally, the ncBAF complex promotes EpiSC gene expression by activating the TGF-β signaling pathway (Fig. 5*A*). Without LIF, both the cBAF and ncBAF complexes work together with the TGF-β pathway to maintain EpiSC gene expression (Fig. 6*B*). Disrupting the cBAF and ncBAF complexes hinders the binding of p-SMAD2 to EpiSC genes, leading to their repression (Fig. 6*B*). The PRC2 complex modulates EpiSC gene expression through two distinct mechanisms. First, it suppresses the LIF/STAT3 signaling pathway, thereby sustaining EpiSC gene expression. Inhibition of PRC2's methyltransferase activity leads to a reduction in EpiSC gene expression upon the inactivation of the LIF/STAT3 pathway. This effect is attributed to the loss of H3K27me3 marks at EpiSC gene loci (Fig. 6*C*). Second, the PRC2 subunit EZH2 enhances p-STAT3, which activates the LIF/STAT3 pathway and subsequently represses EpiSC gene expression (Fig. 6*C*). This research provides the first in-depth investigation into how multiple signaling pathways, including LIF/STAT3, Wnt/β-catenin, TGF-β, the BAF complex, and the PRC2 complex, cooperate to control the transition of mouse ESCs to EpiSCs.

The LIF/STAT3 signaling pathway plays a critical role in maintaining the pluripotency of mouse ESCs (39). Inhibition of STAT3 phosphorylation upon PTPN2 knockdown facilitates the generation of ESC-like colonies in STAT3-overexpressing EpiSCs (40), indicating the repressive function of active STAT3 on the differentiation of ESCs to EpiSCs. In consistent, activation of the LIF/STAT3 pathway drives the reprogramming of EpiSCs to ESCs (10). During the preimplantation-to-postimplantation epiblast transition, the expression of EpiSC genes such as *Pou3f1*, *Fgf5*, *Otx2*, and *Dnmt3a*/*3b* is upregulated, whereas STAT3 is active only in preimplantation blastocysts (41). Inactivation of STAT3 leads to the premature expression of *Otx2* and *Dnmt3a*/*3b*, indicating the necessity of STAT3 to transiently restrict the postimplantation transcriptional program (42). However, the specific regulatory mechanisms by which STAT3 limits this process remain unclear.

Betto *et al.* reported that STAT3 does not regulate Dnmt3a/3b expression through a direct transcriptional mechanism but inhibits their expression through metabolic regulation, thereby maintaining the naive pluripotency of ESCs (42). However, we used multiple methods, including ChIP-seq, ChIP-qPCR, and luciferase reporter assays, to demonstrate the direct regulation of LIF/STAT3 signaling on EpiSC gene expression. Upregulated expression of EpiSC genes, including *Dnmt3a*/*3b*, was observed as early as 6 hours after the depletion of LIF (Fig. 1*C*). Further, LIF starvation, ChIP-qPCR and luciferase experiments support the direct regulation of STAT3 on EpiSC gene expression (Fig. 1, *D*, *F* and *G*). In contrast, Betto *et al.* indicated that STAT3 did not regulate *Dnmt3a*/*3b* expression through a direct transcriptional mechanism (42). The discrepancy between the two studies might be attributed to differences in ChIP-seq data used. Together with the findings reported by Betto *et al.* (42), our results suggest that STAT3 may regulate EpiSC gene expression *via* multiple mechanisms.

Ten Berge *et al.* reported that the Wnt/β-catenin signaling pathway inhibits the transition of naive ESCs to EpiSCs (32). The Wnt/β-catenin and LIF/STAT3 signaling pathways promote the self-renewal of mESCs and reprogram EpiSCs to ESCs through their common target, Sp5 (25). Consistently, this study demonstrates that the LIF/STAT3 and Wnt/β-catenin pathways collaboratively inhibit the expression of EpiSC-specific genes (Figs. 3; S3). Moreover, we discovered that the LIF/STAT3 signaling pathway directly regulates the expression of *Wnt3a* (Figs. 3*C*; S3, *F*–*H*), thereby influencing EpiSC gene expression by controlling the activity of the Wnt/β-catenin signaling pathway. This study highlights the intricate network of signaling pathways involved in maintaining ESC pluripotency and preventing their differentiation into EpiSCs.

The ATP-dependent chromatin remodeling complexes exist in three distinct assemblies: cBAF, polybromo-associated BAF, and the newly characterized ncBAF complexes (13). Both cBAF and ncBAF complexes play crucial roles in maintaining the pluripotency of mouse ESCs (13, 33). BRG1 potentiates LIF signaling by opposing PcG proteins, thereby preventing the differentiation of ESCs (27). Recently, ncBAF was reported to regulate self-renewal in human ESCs by controlling the TGF-β/activin/nodal pathway (34), suggesting its role in the maintenance of mouse EpiSCs. However, the mechanisms by which distinct BAF complexes coordinate with key signaling pathways to regulate the conversion of ESCs to EpiSCs remain unknown. In this study, we revealed that the cBAF complex interacts with the STAT3 and β-catenin pathways to inhibit the expression of EpiSC marker genes, thereby preventing the transition from ESCs to EpiSCs. During the differentiation of ESCs to EpiSCs, the cBAF complex collaborates with the induced TGF-β pathway upon the inactivation of the LIF/STAT3 pathway (Fig. 4*E*), to synergistically promote the expression of EpiSC genes (Figs. 4; S4). In contrast, inhibition of the ncBAF-specific component BRD9 prevented EpiSC gene expression by reducing the activity of the TGF-β/activin/nodal pathway (Figs. 5; S5), thereby inhibiting the transition from ESCs to EpiSCs. Consistent with the promotive role of BRD9 in the maintenance of human ESCs (34), we found that inhibition of BRD9 significantly impaired the maintenance of mouse EpiSCs (Fig. S5*E*). In conclusion, our study elucidates the distinct roles of cBAF and ncBAF complexes in the regulation of TGF-β activity, which underlie their roles in the differentiation of ESCs to EpiSCs.

PRC2 complex represses the differentiation of both mouse and human ESCs *via* catalyzing the methylation of histone H3 at lysine 27 through its enzymatic subunits EZH1 or EZH2 (17, 43, 44). Ho *et al.*, reported that the BAF complex collaborates with the PcG complex to maintain H3K27me3 modification, thereby preventing the differentiation of ESC (27). In this study, we found that the LIF/STAT3 signaling pathway represses the EpiSC gene expression by maintaining the PRC2-mediated H3K27me3 modification, thereby inhibiting the transition from ESC to EpiSC (Fig. 2, *D* and *E*). The inactivation of LIF/STAT3 signaling led to the erase of H3K27me3 modification on EpiSC genes (Fig. 2, *D* and *E*), thereby initiated their expression (Fig. 1*C*). Besides catalyzing H3K27me3, EZH2 has been reported to achieve PRC2-independent activity by methylating non-histone targets or interacting with non-histone proteins to activate downstream genes (45, 46). The PRC2 subunit EZH2 is found to bind and methylate the cardiac transcription factor GATA4, thereby interfering with its transcriptional activity and affecting heart development (45). In glioblastoma stem-like cells, EZH2 binds to and methylates STAT3, leading to enhanced STAT3 activity by increased tyrosine phosphorylation of STAT3 (46). In this study, we revealed for the first time that overexpressing *Ezh2* can activate the LIF/STAT3 signaling pathway in mouse ESCs by increasing p-STAT3 phosphorylation levels independently of its histone methylation activity, thereby inhibiting the expression of EpiSC genes induced upon the withdrawal of LIF (Fig. 2, *B* and *F*). Notably, we have also shown that the inhibition of Ezh2 led to an increase in LIF/STAT3 activity by reducing the H3K27me3 modification at the STAT3 locus and its target genes. So, the dual roles of Ezh2 during the differentiation of ESCs to EpiSCs were first revealed in our study.

## Experimental procedures

### Cell culture

In this study, E14 mouse ESCs (from Sanger Institute) were cultured using two distinct media formulations: 1. fetal bovine serum/LIF medium, which is composed of Dulbecco's modified Eagle medium (DMEM, Procell) enriched with 10% FBS (Excell BIO), 1% penicillin-streptomycin (Gibco), 0.1 mM β-mercaptoethanol (Sigma-Aldrich), 1% L-glutamine (Solarbio), and leukemia inhibitory factor (LIF, GenScript); 2. 2i/LIF medium, which also uses DMEM (Procell) as a base but is supplemented with 10% fetal calf serum (Excell BIO), 1% penicillin-streptomycin (Gibco), 0.1 mM β-mercaptoethanol (Sigma-Aldrich), 1% L-glutamine (Solarbio), along with 3 μM CHIR99021 (Tocris), 1 μM PD0325901, and LIF (GenScript). For both media, the ESCs were maintained on plates coated with 0.1% gelatin (Sigma-Aldrich).

Mouse EpiSCs were cultured in a serum-free medium containing activin A and bFGF, referred to as AF medium, under conditions of 5% CO2 at 37 °C. The AF medium formulation included 20 ng/ml activin A (R&D Systems) and 12 ng/ml bFGF (Thermo Fisher Scientific), supplemented into a base N2B27 medium. To prepare 500 ml of N2B27 medium, 240 ml of DMEM/F12 (Gibco) and 240 ml of Neurobasal (Gibco) were combined with 2.5 ml of N2 supplement (Gibco), 5 ml of B27 supplement (Gibco), 1% L-glutamine (Solarbio), 1% penicillin-streptomycin (Gibco), 1% nonessential amino acids (Gibco), and 0.1 mM β-mercaptoethanol (Gibco). Culture dishes were precoated with recombinant human fibronectin (1 mg/ml in Dulbecco's phosphate buffered saline, East Mab Bio) for at least 24 h before use. Mouse EpiSCs were routinely passaged every 2 days using Accutase (Chemicon & Upstate) for cell dissociation, at a ratio of 1:3 to 1:6. Fresh AF medium was replenished daily to maintain stable cell growth.

### Generation of STAT3-ERT2 mouse ESCs

To generate the STAT3-ERT2 cell line, a cotransfection method was used, wherein 2 μg of the PB-STAT3-ERT2 plasmid and 0.7 μg of transposase plasmid were concurrently introduced into ESCs. Following transfection, the cells were cultured with 100 μg/ml hygromycin for 5 days to select for stable transfectants. The construction of the cell line was validated through quantitative PCR and Western blot analysis.

### Quantitative RT-PCR

Quantitative RT-PCR was performed using a ViiA7 real-time PCR system (Applied Biosystems) following a 3-step protocol. Total RNA was isolated using the FastPure Cell/Tissue Total RNA Isolation Kit V2 (Vazyme), and complementary DNA was synthesized with the HiScript II Q RT SuperMix (Vazyme). The PCR conditions included an initial denaturation step at 95 °C for 30 s, followed by 40 cycles of denaturation at 95 °C for 10 s and annealing/extension at 60 °C for 40 s. Real-time PCR reactions were conducted with Taq Pro Universal SYBR qPCR Master Mix (Vazyme), and gene expression levels were normalized to Gapdh transcript levels. qRT-PCR data were analyzed by GraphPad prism (https://www.graphpad.com/). Error bars represent the standard deviation of three technical replicates from a representative experiment. Primer sequences used for qPCR analysis are detailed in Table S1.

### Western blot analysis

Protein samples were fractionated on 10% SDS-PAGE gels, electroblotted onto polyvinylidene fluoride membranes (Millipore), and membranes probed sequentially with respective antibodies. The blots were incubated with secondary antibodies, developed using ECL Plus (Epizyme Biotech), and subsequently imaged with a ChemiScope S6 imaging system (Clinx Science Instruments Co, Ltd). The antibody information was provided in Table S1.

### Co-immunoprecipitation

Cells were lysed using a lysis buffer composed of 1% IGEPAL CA-630 (Sigma-Aldrich), 1 × protease inhibitor (Sigma-Aldrich), 50 mM Tris–HCl (pH 8.0), and 150 mM NaCl. Following lysis, one-tenth of the lysate was retained as input, while the remaining supernatant was subjected to preclearance with Protein A/G beads at 4 °C for 30 min. The resultant supernatant was then incubated at 4 °C for 2 h with the target-specific antibody and an isotype control, after which Protein A/G beads were added, followed by overnight incubation at 4 °C. The resulting co-immunoprecipitation complex was purified and subsequently denatured by boiling with 2.5 × protein loading buffer for 10 min to prepare for Western blot analysis.

### Fgf5/Otx2/Dnmt3a promoter constructs for luciferase assay

The 5′-flanking region of Fgf5 was amplified using the primer set: CTAGCTAGCAGAAGTAACAATCTTTCTCCT and CTAAAGCTTACTATTCCCTTCATACACAAA, generating a 1344 bp fragment upstream of the first ATG. This fragment contained NheI and HindIII restriction sites at each end, respectively. Similarly, the 5′-flanking region of Otx2 was amplified with primers CTAGCTAGCGTAGGAATGCACCCCT and CTAAAGCTTTCCCAGCCTCTTGTTCCC, resulting in a 1062 bp fragment upstream of the first ATG, also incorporating NheI and HindIII sites at each end. The 5′-flanking region of Dnmt3a was amplified using the primers CTAGCTAGCACCTCAGTGCCTTTAGGATAT and CTAAAGCTTACAGATTAGAGATGAGGCTG, yielding a 1163 bp fragment upstream of the first ATG, with NheI and HindIII sites at each end. Mouse genomic DNA isolated from ESCs was used as the template for amplifying these 5′-flanking regions. After digestion with NheI (NEB) and HindIII (NEB), the fragments were cloned into the promoterless pGL3-Basic luciferase reporter plasmid (Promega) to generate the Fgf5/Otx2/Dnmt3a luciferase reporter constructs.

### Gene transfection

Transfection was conducted using Lipofectamine 8000 transfection reagent (Beyotime) in accordance with the manufacturer's guidelines. In summary, ESCs were cultivated in six-well culture plates, and subsequently transfected with the DNA:lipofectamine complex.

### Luciferase assay

Following a 30-h transfection period with the Fgf5/Otx2/Dnmt3a luciferase reporter construct, luciferase activity of the reporter genes was quantified using the dual luciferase assay kit protocol (Vazyme). Briefly, the culture medium was removed, and cells were rinsed twice with cold PBS. Subsequently, 100 to 150 μl of passive lysis buffer was added to each well, and the cells were incubated at 37 °C for 10 min before being transferred to 96-well plates. To measure luminescence, 25 μl of luciferase substrate (LAR II) was added. Following this, 25 μl of Stop & Glo reagent was introduced to terminate the reaction, allowing for the measurement of the internal standard Renilla luciferase. Relative luciferase activity was normalized to the internal Renilla luciferase activity.

### RNA-seq analysis

Total RNA was extracted using the Total RNA Isolation Kit. Subsequent sequencing was performed on Illumina HiSeq 2500 machines. The raw reads obtained were aligned to the reference genome (version mm10) using HISAT2. Gene count tables were generated using HTSeq with default parameters. Differential gene expression analysis was conducted with DESeq2, also using default parameters. The complete list of differentially expressed genes is provided in Table S2. Heatmap visualizations of various sets of differentially expressed genes were created using pheatmap (v1.0.12; https://cran.r-project.org/package=pheatmap) and ComplexHeatmap (v2.14.0; https://bioconductor.org/packages/release/bioc/html/ComplexHeatmap.html).

### GO analysis

GO analysis for enriched biological processes was performed using Metascape (http://metascape.org) to find significantly enriched terms.

### ChIP-seq and ChIP-qPCR experiments

ChIP-seq and ChIP-qPCR experiments were performed as previously described (17, 47). Briefly, approximately 1 × 10ˆ7 cells were fixed with 1% formaldehyde at room temperature for 10 min, followed by quenching with 0.125 M glycine. The cross-linked cells were resuspended in sonication buffer and sonicated using a Bioruptor. Sonication included three rounds with low mode pulsing (30s ON; 30s OFF), followed by ten rounds with high mode pulsing (30s ON; 30s OFF). A one-thirtieth fraction of the sonicated chromatin was reserved as input. The remaining chromatin was incubated with 1 μg of antibody conjugated to magnetic beads overnight at 4 °C. After immunoprecipitation, the beads were sequentially washed with radioimmunoprecipitation assay buffer, low salt buffer, high salt buffer, LiCl buffer, and 1 × TE buffer. DNA libraries were generated using Tn5 transposase. Subsequently, DNA was extracted by reversing crosslinks at 65 °C overnight with proteinase K (20 μg/ml) and then sequenced on the Illumina NovaSeq 6000 platform.

ChIP-qPCR was conducted following established protocols (47). In brief, ChIP assays were performed as previously described, excluding the DNA library preparation step. Following the purification of immunoprecipitated DNA, 1 μl of the sample was used for each qPCR reaction. The qPCR was conducted using the Taq Pro Universal SYBR qPCR Master Mix (2 × ). Each qPCR was performed in duplicate, based on at least two independent experiments. Data were normalized to input values and calculated as the fold change relative to input. The ChIP-qPCR primers were designed based on ChIP-seq results and are listed in Table S1.

### Quantification and statistical analysis

The statistical information for each experiment, including the total number of samples analyzed and the specific statistical tests, is presented as mean ± SEM of three independent experiments unless otherwise stated. *p*-values were calculated using Mutiple *t* test, and statistical significance was considered when *p* < 0.05 (∗), *p* < 0.01 (∗∗), *p* < 0.001 (∗∗∗), *p* < 0.001 (∗∗∗∗).

### ChIP-seq data analysis

Firstly, we used fastp (version 0.20.0) to preprocess the sequencing data in FASTQ format, using the "-l 25 --detect_adapter_for_pe" options for adapter trimming and quality filtering. Then, we used HISAT2 (version 2.2.1) with the "--no-temp-splicesite --no-spliced-alignment" parameters to align the reads to the mouse mm10 reference genome. Next, we used samtools with the "-f2 -F3844 -q 30" options to filter out reads with a mapping quality below 30 and those that were not properly paired. After that, we used sambamba with the "-r" parameter to remove duplicate reads. Subsequently, we applied MACS2 (version 2.2.7.1; https://pypi.org/project/MACS2/) with the "-q 0.05 --keep-dup all -f BAMPE -g mm" options to perform peak calling on immunoprecipitation and input samples, in order to identify enriched regions in the genome.

Finally, we used bedGraphToBigWig to convert the bedGraph files into BigWig format, which were then uploaded to the UCSC Genome Browser for visualization. All commands can be found in the Github repository: https://github.com/yilia2023/LIF-STAT3-Regulates-H3K27me3-deposition_Chip-seq_analysis.

### Differential analysis

To analyze the differential levels of H3K27me3 modifications in ESCs under control and no LIF conditions, we used the mergeBed tool from the bedtools suite to combine narrowPeak files and create a union peak set by merging peaks with at least a one bp overlap. Then, we used coverageBed from the same suite to count the mapped reads across these union peaks for each condition. Differential modification of H3K27me3 genomic sites with at least 2-fold change between control and no LIF samples was determined using DESeq2 (version 1.38.3; https://bioconductor.org/packages/release/bioc/html/DESeq2.html) in R (version 4.2.0; https://www.r-project.org/), applying the default false discovery rate adjustment for multiple hypothesis testing. The threshold for identifying differential sites was set at Padj < 0.05 and |log2FoldChange| > =1. The exact code for the differential analysis can be found in this GitHub repository: https://github.com/yilia2023/LIF-STAT3-Regulates-H3K27me3-deposition_Chip-seq_analysis.

### Enrichments analysis

To generate Figure 2*C* and S2*C*, we used bamCoverage from the deepTools suite to produce coverage profiles of mapped reads, normalized using the --normalizeUsing RPKM option. Then, we used computeMatrix from the deepTools suite to calculate the enrichment relative to the summits of H3K27me3 peaks, operating in reference-point mode with the parameters --referencePoint center -b 8000 -a 8000 -bs 100. Finally, we visualized the output matrix using plotHeatmap and plotProfile from deepTools. To generate Fig. S2*D*, we performed an enrichment analysis of biological processes using GREAT (http://great.stanford.edu/public/html/), focusing on identifying terms with significant enrichment (*p* value < 0.01) within the genomic regions associated with H3K27me3 modifications.

To generate Figure 3*G*, we use the matplotlib-venn package in Python to draw Venn diagrams (https://pypi.org/project/matplotlib-venn/). The codes can be found in the GitHub repository: https://github.com/yilia2023/LIF-STAT3-Regulates-H3K27me3-deposition_Chip-seq_analysis.

## Data availability

The RNA-Seq and ChIP-seq raw data of this study were deposited in the NCBI BioProject database under the project ID PRJNA1145863.

## Supporting information

This article contains supporting information.

## Conflict of interest

The authors declare that they have no conflict of interest with the contents of the article.
