# Supplementary material for: Interplay of chromatin remodeling BAF complexes in mouse embryonic and epiblast stem cell conversion and maintenance
Source: J Biol Chem. 2024 Dec 25;301(2):108140. doi: 10.1016/j.jbc.2024.108140 (PMC11791114; doi:10.1016/j.jbc.2024.108140)
Supplement: Supplementary Figures and Tables [file mmc1.docx]

**Figure S1. The cBAF complex collaborates with the LIF/STAT3 pathway to repress the transition ESCs to EpiSCs.**

(A) qPCR analysis of transcript levels for *Fgf5*, *Otx2*, *Dnmt3a* and *Lef1* in WT and *Dpf2* KO ESCs. (B) qPCR analysis of transcript levels for *Oct4*, *Sox2*, *Nanog*, *Klf4* and *Esrrb* genes in ESCs cultured in ESC medium without LIF for 24 hours. (C) qPCR analysis of transcript levels for *Fgf5*, *Otx2*, *Dnmt3b*, *Dnmt3a*, and *Pou3f1* in ESCs cultured in ESC medium, ESC medium without LIF for 24 hours, followed by an additional 24 hours with and without LIF;

(D) Schematic diagram of STAT3-ERT2 ESCs. (E) Western blot analysis depicting protein levels of STAT3 and p-STAT3 in STAT3-ERT2 ESCs cultured in ESC medium in the absence of LIF, with and without 1μM of 4-OHT treatment for 48 hours, GAPDH was used as a loading control. (F) qPCR analysis of transcript levels for the genes *Nanog*, *Tbx3*, *Mras*, *Eya1*, *Stat3*, *Gjb3*, *Lama1*, *Esrrb*, *Fabp3* and *Ppap2b* in STAT3-ERT2 ESCs cultured in ESC medium in the absence of LIF, with and without 1μM of 4-OHT treatment for 48 hours. (G) qPCR analysis of transcript levels for *Fgf5*, *Otx2*, *Dnmt3b*, *Dnmt3a*, *Lef1* and *Pou3f1* in ESCs cultured in ESC medium without LIF for 24 hours, followed by an additional 24 hours without LIF, with and without 1μM of 4-OHT treatment. (H) ChIP-qPCR analysis of STAT3 levels at the promoter regions of the *Fgf5*, *Otx2*, *Dnmt3b*, *Dnmt3a*, *Lef1* and *Pou3f1* genes in ESCs cultured in ESC medium with and without LIF for 48 hours. (I) qPCR analysis of transcript levels for *Fgf5*, *Otx2*, *Dnmt3a*, *Dnmt3b*, *Lef1*, and *Pou3f1* in ESCs cultured in N2B27 medium with 2i, AF medium with and without LIF. (J) qPCR analysis of transcript levels for *Mras*, *Ly6g6e*, *Cobl*, *Gjb3*, *Lrrc34*, *Fabp3*, and *Lama1* in *Dpf2^fl/fl^* (WT) and *Dpf2* KO ESCs. * indicates p < 0.05, ** indicates p < 0.01, *** indicates p < 0.001, **** indicates p < 0.001. Error bars represent the standard deviation.

**Figure S2. Collaborative Repression of ESC to EpiSC Transition by cBAF, PRC2 Complexes, and the LIF/STAT3 Pathway.** (A) qPCR analysis of transcript levels for *Ezh2*, *Eed*, and *Suz12* in ESCs cultured in ESC medium, ESC medium without LIF, and ESC medium with LIF re-addition after LIF starvation. (B) qPCR analysis of transcript levels for *Fgf5*, *Otx2*, *Dnmt3a*, *Dnmt3b*, and *Pou3f1* in ESCs cultured in ESC medium without LIF, with and without *Eed* overexpression for 48 hours. (C) Heatmap representation of normalized tag density profiles depicting increased H3K27me3 in ESCs cultured with and without LIF, along with corresponding metaplots illustrating signal intensities. (D) GO analysis for biological processes associated with the genes located in the vicinity of the reduced and gained H3K27me3 sites upon the depletion of LIF. (E) Genome browser view of ChIP-seq tracks for H3K27me3 at the *Mras*, *Tbx3* and *Gjb3* loci in ESCs cultured with (Ctrl.) and without LIF (No LIF). (F-G) qPCR analysis of transcript levels for *Stat3*, *Ly6g6e*, *Esrrb*, *Mras*, and *Tbx3* in ESCs cultured in ESC medium in the absence of LIF, with and without overexpression of *Ezh2* (F) or *Eed* (G) for 48 hours. (H) Heatmap illustrating the upregulated genes in the absence of LIF, which are downregulated upon 10μM of GSK126 treatment, identified through RNA-seq analysis in ESCs cultured in ESC medium (Ctrl.), ESC medium without LIF (-LIF), and ESC medium without LIF with 10μM of GSK126. (I) GO analysis for biological processes associated with genes differentially expressed identified in (H). (J) qPCR analysis of transcript levels for *Tbx3*, *Mras*, *Eya1*, *Ly6g6e*, *Esrrb*, *Stat3*, *Gjb3* and *Fabp3* in ESCs cultured in ESC medium in the absence of LIF, with and without 10μM of GSK126. * indicates p < 0.05, ** indicates p < 0.01, *** indicates p < 0.001, **** indicates p < 0.001. Error bars represent the standard deviation.

**Figure S3. LIF/STAT3 pathway inhibits the expression of EpiSC genes via maintaining the activity of Wnt pathway.** (A) qPCR analysis of transcript levels for *Fgf5*, *Otx2*, *Dnmt3a*, *Dnmt3b*, *Pou3f1*, and *Lef1* in ESCs cultured in ESC medium, with and without 3µM of CHIR99021 activator for 48 hours. (B) qPCR analysis of transcript levels for *Fgf5*, *Otx2*, *Dnmt3a*, *Dnmt3b*, and *Pou3f1* in ESCs cultured in 2i ES medium (Ctrl.) and 2i medium without 3 µM CHIR99021 for 48 hours. (C) qPCR analysis of transcript levels for *Fgf5*, *Otx2*, *Dnmt3a*, *Dnmt3b*, *Lef1*, and *Pou3f1* in ESCs cultured in 2i ESC medium with and without LIF, and in 2i medium without 10μM of CHIR99021 with and without LIF. (D) Morphology of ESCs cultured in standard ESC medium, ESC medium without LIF, and ESC medium without LIF plus 10μM of CHIR99021. The scale bar represents 200 μm. (E) qPCR analysis of transcript levels for *Ctnnb1* in ESCs cultured in ESC medium without LIF for the specified duration. (F) qPCR analysis of transcript levels for *Wnt3a* in STAT3-ERT2 ESCs cultured in ESC medium (Ctrl.), in ESC medium without LIF, with and without 1μM of 4-OHT for 48 hours. (G) Genome browser view of ChIP-seq tracks forH3K27me3 at the Wnt3a loci in ESCs cultured in ESC medium with (Ctrl.) and without LIF (No LIF). (H) ChIP-qPCR analysis of H3K27me3 levels at the promoter regions of the *Wnt3a* in ESCs cultured in ESC medium with and without LIF for 48 hours. (I) Genome browser view of ChIP-seq tracks for β-CATENIN and STAT3 at the *Lef1* and *Dnmt3a* loci in ESCs. (J) GO analysis for biological processes associated with the common target genes of DPF2, β-CATENIN, and STAT3. * indicates p < 0.05, ** indicates p < 0.01, *** indicates p < 0.001, **** indicates p < 0.001. Error bars represent the standard deviation.

**Figure S4. The cBAF complex collaborates with the TGF-β pathway to maintain the expression of EpiSC genes.**

(A) Morphology of EpiSCs cultured in AF medium and passaged 1 to 3 times with BRM/BRG1 ATP inhibitor 1 treatment. The scale bar represents 150 μm. (B) ChIP-qPCR analysis of BRG1 levels at the promoter regions of the *Gjb3*, *Eya1*, *Mras*, and *Lama1* genes in ESCs cultured in ESC medium with and without LIF for 48 hours. (C) ChIP-qPCR analysis of H3K27me3 levels at the promoter regions of *Fgf5*, *Otx2*, *Dnmt3b*, *Dnmt3a*, *Lef1*, and *Pou3f1* in ESCs cultured in ESC medium with (Ctrl.) and without LIF (-LIF) for 48 hours, and in *Dpf2* KO ESCs cultured in ESC medium without LIF (*Dpf2^-/-^*, -LIF). (D) qPCR analysis of transcript levels for *Lefty1*, *Lefty2*, *Pitx2*, and *Nodal* in *Dpf2^fl/fl^* ESCs cultured in ESC medium with the addition of 4-OHT for the specified duration. (E-F) Genome browser view of ChIP-seq tracks depicting BRG1, DPF2, β-CATENIN, and STAT3 binding in WT ESCs, SMAD2/3 binding in WT ESCs, and Day 3 EBs induced with Activin A (ACT) at the *Lef1* (E) and *Dnmt3a* (F) loci. (G) ChIP-qPCR analysis of SMAD2 levels for *Fgf5*, *Otx2*, and *Dnmt3b* in WT ESCs cultured in ESC medium with and without LIF, and in *Dpf2* KO ESCs cultured in ESC medium without LIF for 96 hours. * indicates p < 0.05, ** indicates p < 0.01, *** indicates p < 0.001. Error bars represent the standard deviation.

**Figure S5. ncBAF complex represses the transition of ESCs to EpiSCs.** (A) qPCR analysis of transcript levels for *Fgf5*, *Otx2*, *Dnmt3a*, *Dnmt3b*, *Lef1*, and *Pou3f1* in ESCs cultured in ESC medium, ESC medium upon LIF depletion, with and without 10μM of I-BRD9. (B) Western blot analysis depicting protein levels of STAT3 in ESCs cultured in ESC medium with and without 10μM of I-BRD9 treatment. GAPDH was used as a loading control. (C) qPCR analysis of transcript levels for *Gjb3*, *Mras*, *Eya1*, *Stat3*, *Lama1*, *Ly6g6e*, and *Fabp3* in ESCs cultured in ESC medium with and without 10μM of I-BRD9 treatment. (D) Western blot analysis depicting protein levels of SMAD2 in ESCs cultured in ESC medium with and without 10μM of I-BRD9 treatment. GAPDH was used as a loading control. (E) Morphology of EpiSCs cultured in AF medium and passaged 1 to 3 times with 10μM of I-BRD9 treatment. The scale bar represents 150 μm. * indicates p < 0.05, ** indicates p < 0.01, *** indicates p < 0.001. Error bars represent the standard deviation.

**Table S1**. Primer sequences for qPCR, ChIP-qPCR, and antibody information for experimental use.

**Table S2**. Genes associated with co-binding sites of DPF2, β-CATENIN, and STAT3 identified through ChIP-seq analysis.
