# Supplementary material for: Interplay of chromatin remodeling BAF complexes in mouse embryonic and epiblast stem cell conversion and maintenance
Source: J Biol Chem. 2024 Dec 25;301(2):108140. doi: 10.1016/j.jbc.2024.108140 (PMC11791114; doi:10.1016/j.jbc.2024.108140)

Figure S1 The cBAF complex collaborates with the LIF/STAT3 pathway to repress the transition ESCs to EpiSCs.

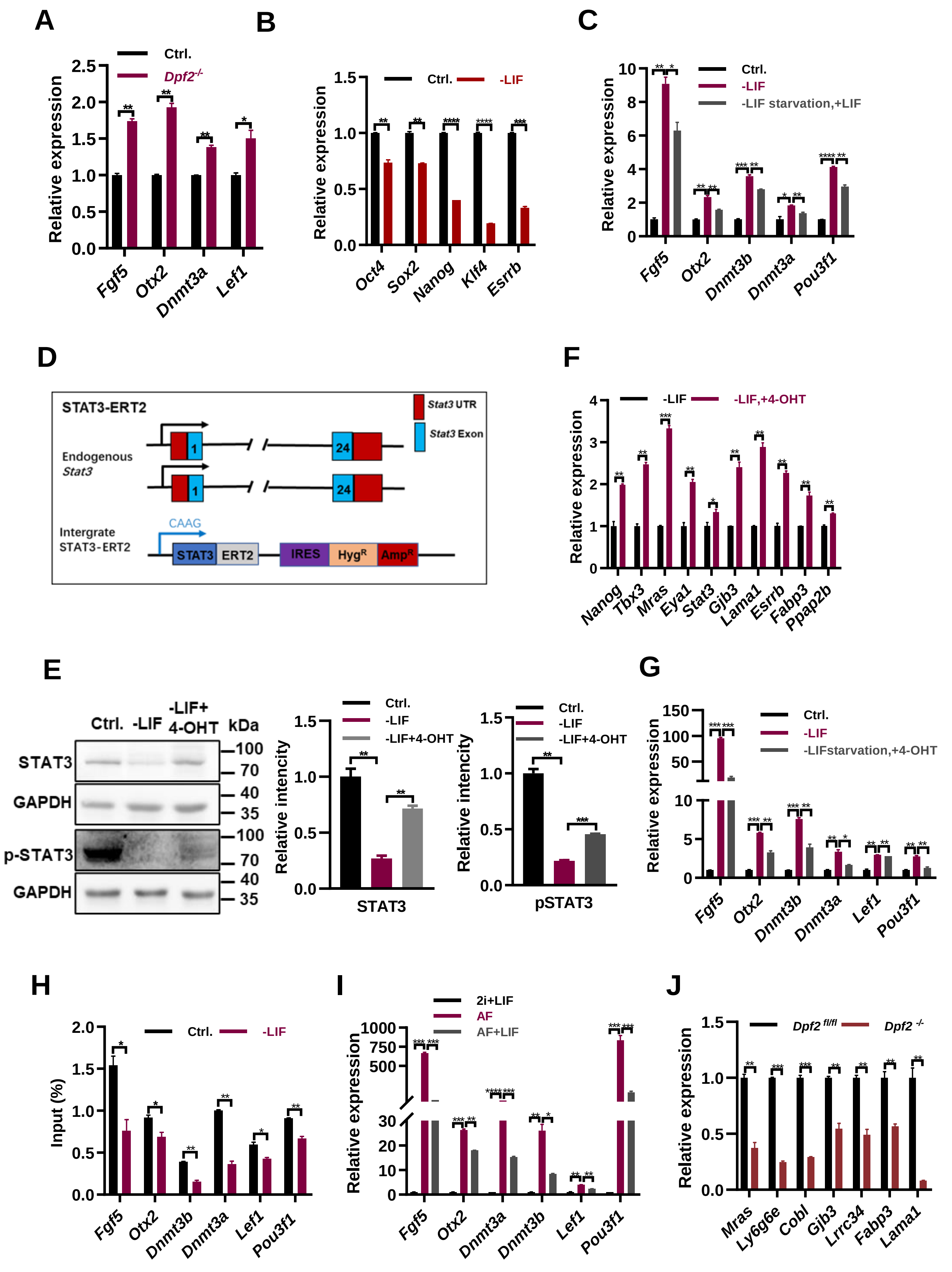

Figure 2 Collaborative Repression of ESC to EpiSC Transition by cBAF, PRC2 Complexes, and the LIF/STAT3 Pathway.

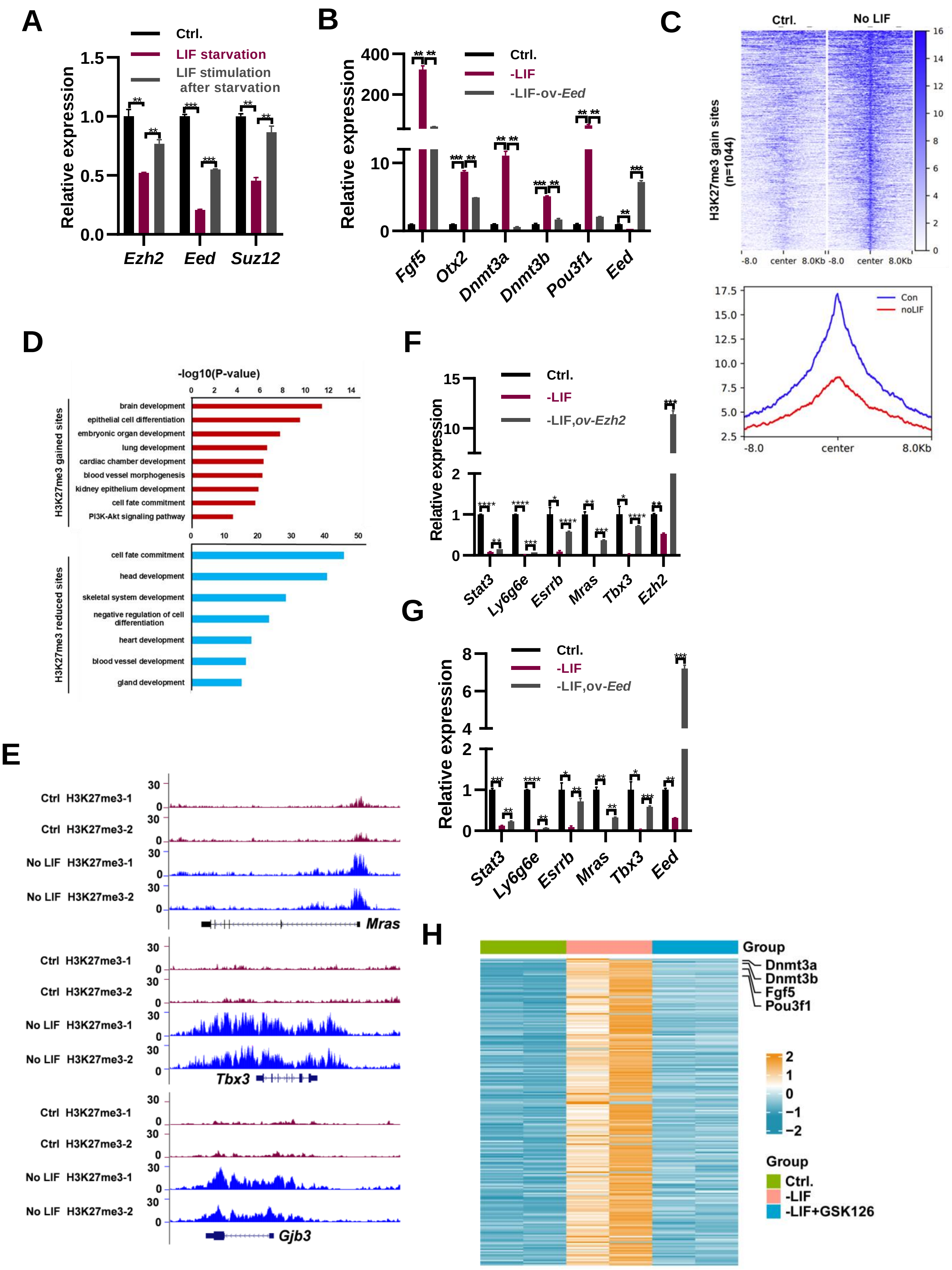

Figure 2 Collaborative Repression of ESC to EpiSC Transition by cBAF, PRC2 Complexes, and the LIF/STAT3 Pathway.

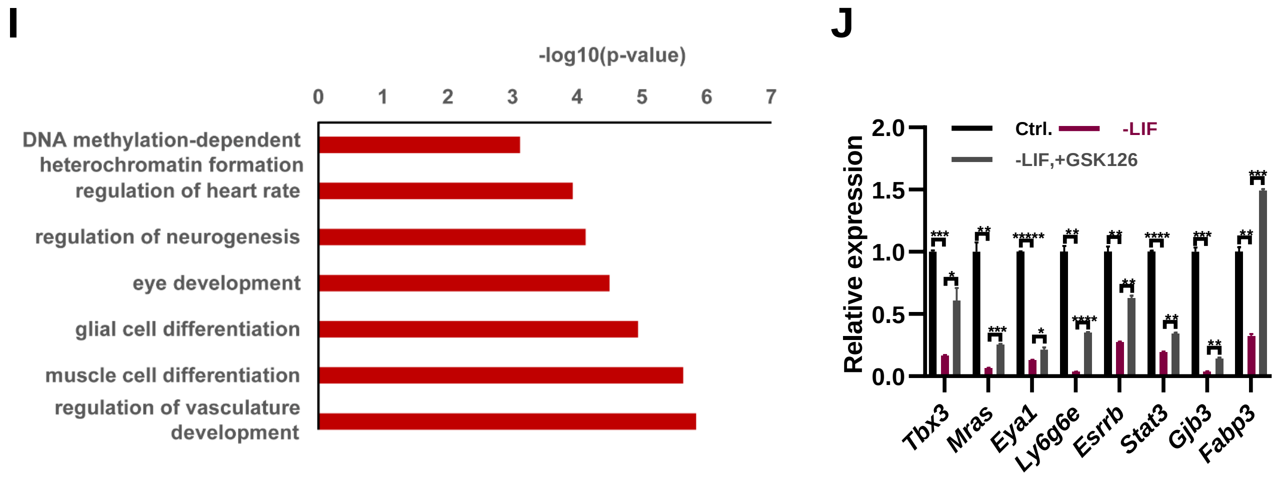

Figure S3. LIF/STAT3 pathway inhibits the expression of EpiSC genes via maintaining the activity of Wnt pathway.

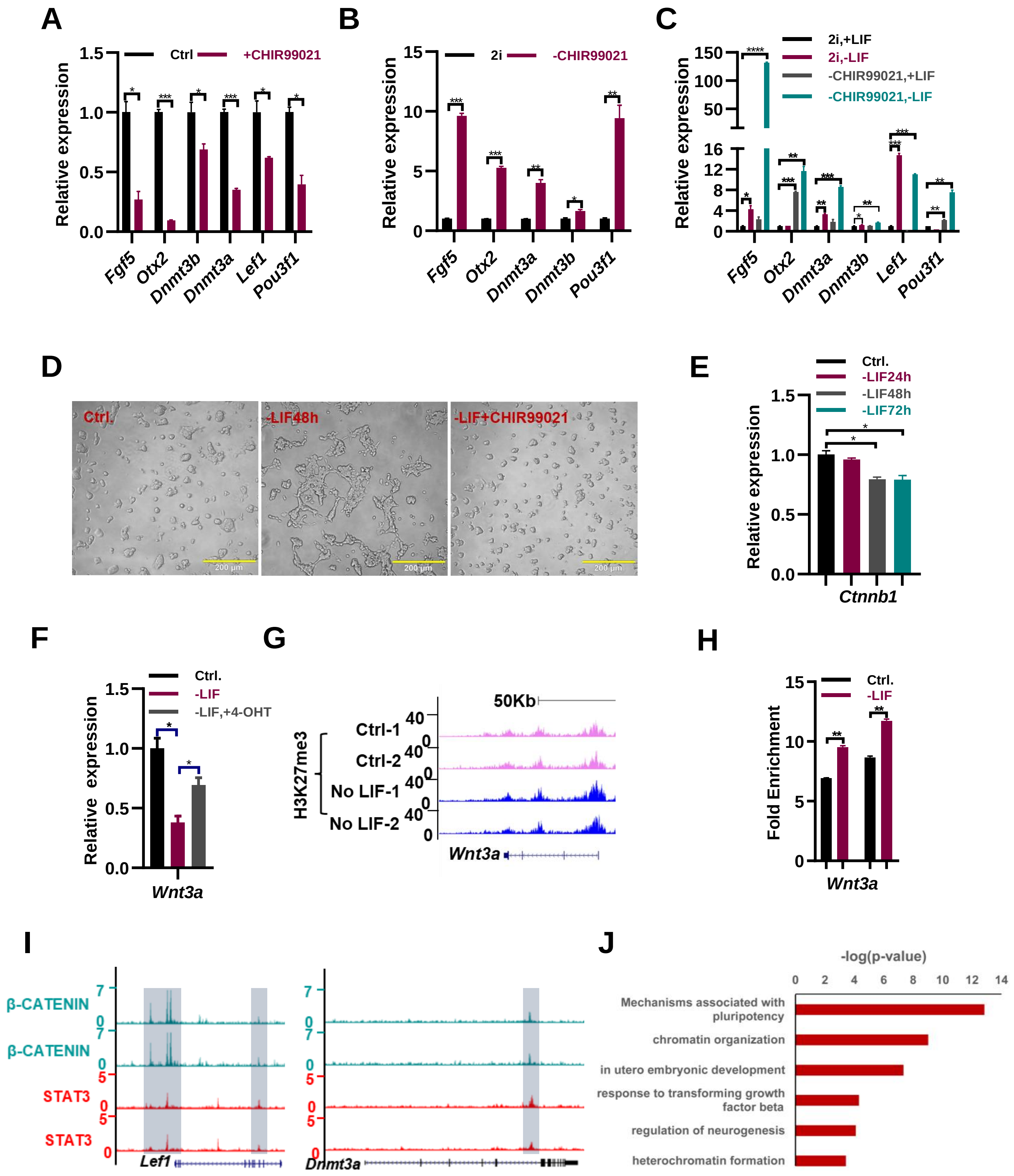

Figure S4. cBAF complex collaborative with TGF-β pathway to maintain the expression of EpiSC genes.

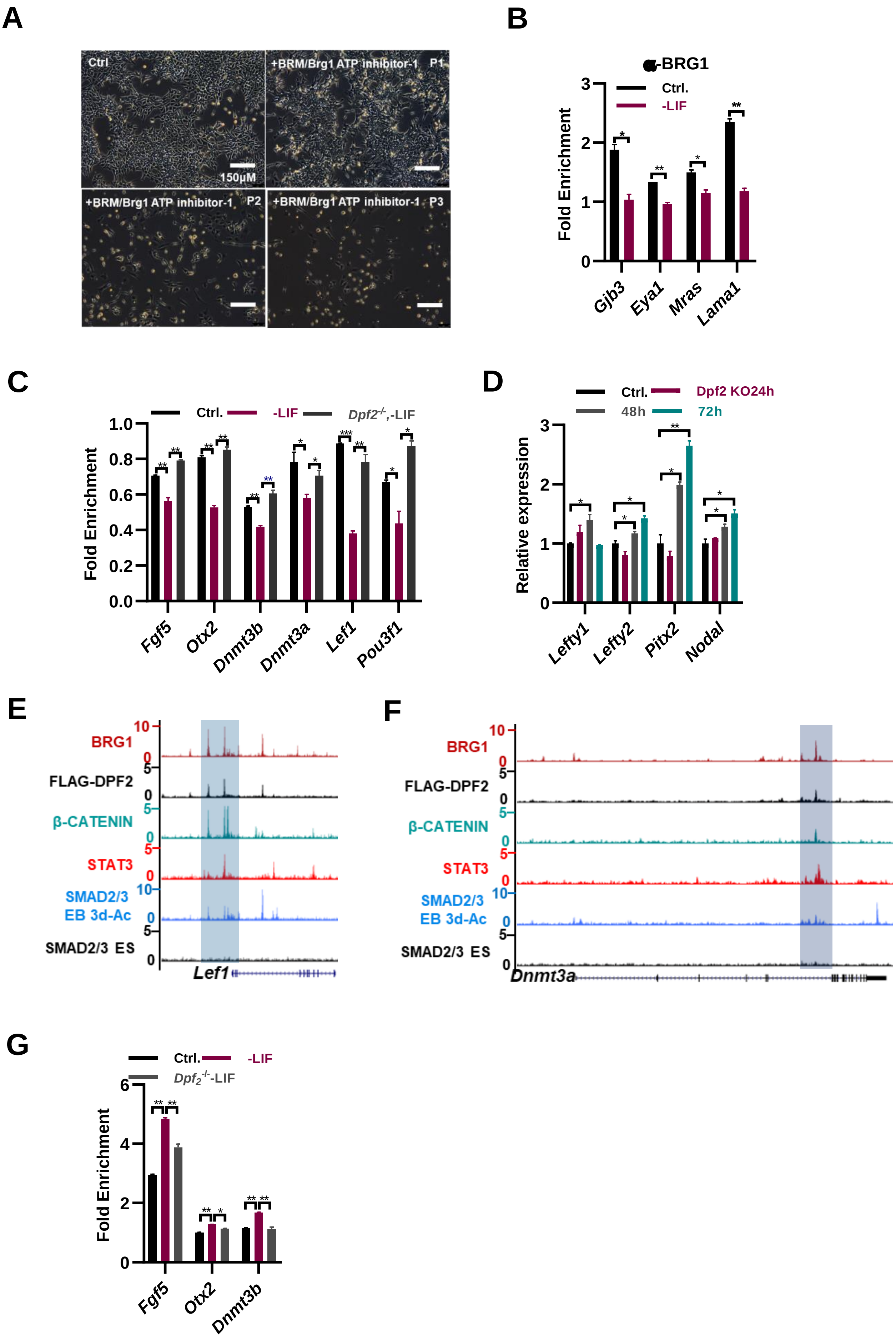

Figure S5. ncBAF complex represses the transition of ESCs to EpiSCs.

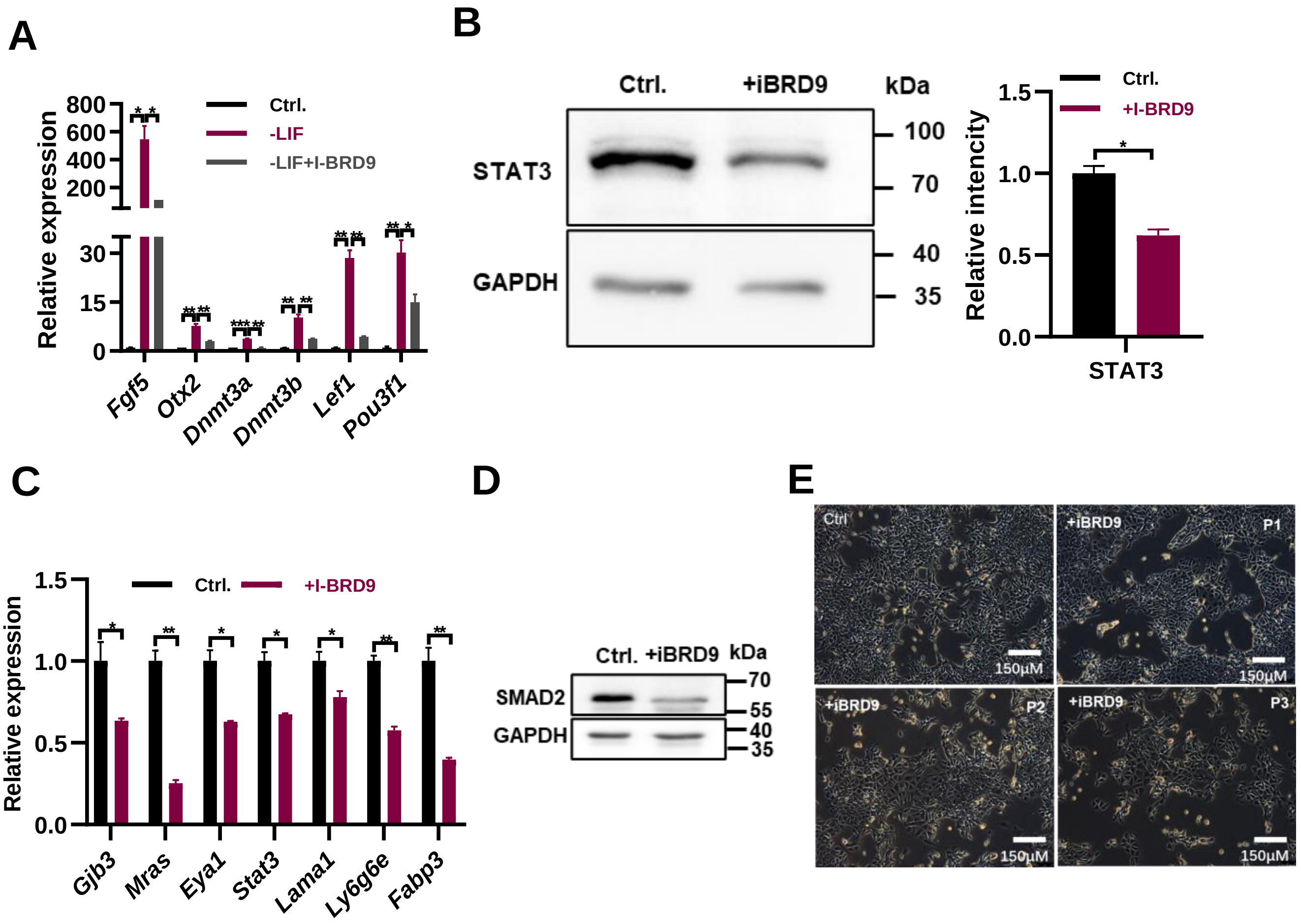

Supplement: Supplementary Figures [file mmc2.pdf]
